# MK3 Modulation Affects BMI1-Dependent and Independent Cell Cycle Check-Points

**DOI:** 10.1371/journal.pone.0118840

**Published:** 2015-04-08

**Authors:** Peggy Prickaerts, Hanneke E. C. Niessen, Vivian E. H. Dahlmans, Frank Spaapen, Juliette Salvaing, Jolien Vanhove, Claudia Geijselaers, Stefanie J. J. Bartels, Iris Partouns, Dietbert Neumann, Ernst-Jan Speel, Yoshihiro Takihara, Bradly G. Wouters, Jan Willem Voncken

**Affiliations:** 1 Department of Molecular Genetics, Maastricht University Medical Centre, Maastricht, the Netherlands; 2 Department of Pathology, Maastricht University Medical Centre, Maastricht, the Netherlands; 3 Department of Stem Cell Biology, Research Institute for Radiation Biology and Medicine, Hiroshima University, Hiroshima, Japan; 4 Princess Margaret Cancer Centre and Campbell Family Institute for Cancer Research, Departments of Radiation Oncology and Medical Biophysics, University Health Network, Toronto, Canada, Maastricht Radiation Oncology (MaastRO) Lab, Maastricht University, Maastricht, The Netherlands; University of Montréal and Hôpital Maisonneuve-Rosemont, CANADA

## Abstract

Although the MK3 gene was originally found deleted in some cancers, it is highly expressed in others. The relevance of MK3 for oncogenesis is currently not clear. We recently reported that MK3 controls ERK activity via a negative feedback mechanism. This prompted us to investigate a potential role for MK3 in cell proliferation. We here show that overexpression of MK3 induces a proliferative arrest in normal diploid human fibroblasts, characterized by enhanced expression of replication stress- and senescence-associated markers. Surprisingly, MK3 depletion evokes similar senescence characteristics in the fibroblast model. We previously identified MK3 as a binding partner of Polycomb Repressive Complex 1 (PRC1) proteins. In the current study we show that MK3 overexpression results in reduced cellular EZH2 levels and concomitant loss of epigenetic H3K27me3-marking and PRC1/chromatin-occupation at the *CDKN2A/INK4A* locus. In agreement with this, the PRC1 oncoprotein BMI1, but not the PCR2 protein EZH2, bypasses MK3-induced senescence in fibroblasts and suppresses P16^INK4A^ expression. In contrast, BMI1 does not rescue the MK3 loss-of-function phenotype, suggesting the involvement of multiple different checkpoints in gain and loss of MK3 function. Notably, MK3 ablation enhances proliferation in two different cancer cells. Finally, the fibroblast model was used to evaluate the effect of potential tumorigenic MK3 driver-mutations on cell proliferation and M/SAPK signaling imbalance. Taken together, our findings support a role for MK3 in control of proliferation and replicative life-span, in part through concerted action with BMI1, and suggest that the effect of MK3 modulation or mutation on M/SAPK signaling and, ultimately, proliferation, is cell context-dependent.

## Introduction

Sequential activation of kinases within the canonical M/SAPK (mitogen/stress activated protein kinase) cascades is a common and evolutionary-conserved signal transduction mechanism. The canonical M/SAPK cascades cooperate in transmitting and integrating intra- and extracellular signals, thereby controlling a large number of, sometimes opposing, cellular processes such as proliferation, differentiation, survival, stress response, and apoptosis [1]. Downstream of M/SAPKs, MAPK-activated protein kinases (MAPKAPKs), including RSK1-4, MSK1/2, MNK1/2 and MK2/3/5, signal to diverse cellular targets. Among the MAPKAPKs are three structurally related MKs MK2, MK3 and MK5. Despite their high homology, the three MKs display distinct spatio-temporal expression profiles and act in different biological processes [2,3]. Identification of MK-substrates suggests that MKs function in numerous cellular processes, including gene transcription, mRNA-stability and translation, cytoskeleton remodeling, cell proliferation and apoptosis. Besides their joint involvement in inflammatory responses, the biological relevance of substrate interaction and phosphorylation by MKs remains largely unclear.

MK3 (MAPKAPK3, 3pK) was identified as the first MK activated down-stream of all three mitogen- and stress-activated protein kinase (M/SAPK) cascades; consequentially, MK3 was considered an integration point of converging mitogenic and stress signaling [4]. Whereas the RAS-M/SAPK signalling pathways have a long-standing link to cancer, the involvement of MKs in cancer is currently unclear. MK3, originally referred to as 3pK (chromosome 3p kinase), was found frequently homozygously deleted as part of the 3p21.3 region in small cell lung cancer and other cancers and cancer cell lines [5,6]. Conversely, potential oncogenic ‘driver’ mutations have been identified in MK3 [7]. These records point to an involvement of MK3 in tumorigenesis and support the idea that it may act tumorigenic or tumor-suppressive.

We previously reported that MK3 associates with PRC1-complexes through direct SAM (Self-Association Motif) domain-mediated interaction with the *Polyhomeotic* orthologs PHC1 and PHC2 [8]. Polycomb Group repressive complexes (PRC1 and PRC2) act as part of a cellular epigenetic memory system and play an important role in the determination of cell fate [9]. Both core complexes harbor intrinsic PRC protein-associated epigenetic catalytic activity, and are known to interact with additional epigenetic regulators. These interactions and catalytic activities are controlled by post-translational modification [10,11]. In addition, we established that phosphorylation of the PRC1 complex controls PRC1/chromatin-association [8,12]. PRC proteins have been linked to oncogenesis: high expression or mutation of several PRC members has been etiologically implicated in the onset and malignant progression of cancer [13].

Recent data from our group identified MK3 as a regulator of the PRC1 target gene ATF3-expression via a negative feedback mechanisms on MEK1 and ERK1/2 in the context of mitognic stimulation. We found that MK3 ablation or inhibition resulted in prolonged ERK phosphorylation and increased ATF3 and premature and elevated EGR1 expression [14]. Using a genetic *Drosophila* model for wing development, we confirmed exagerated mitogenic ERK-signaling in the absence of dMK (the only *Drosophila* MK ortholog), thus supporting a negative regulatory role for MK3 in canonical ERK signaling [14]. In addition, besides ERK, modulation of MK3 levels also affects cellular P38 and JNK protein levels [14,15]. Combined, these observations suggest that altered cellular MK3 levels and or activity may contribute to deregulation of cellular proliferation through deregulation of M/SAPK, potentially contributing to tumorigenesis.

In this study we tested the hypothesis that MK3 controls cell proliferation using MK3-gain- and loss-of-function constructs in normal and cancer cells. In addition, we aimed to investigate the biological relevance of the interaction between MK3 and PRC1 in the context of cell proliferation. We provide evidence for a genetic interaction between the Polycomb Group protein BMI1 and MK3 in proliferative life span: BMI1 overcame the MK3-overexpression phenotype in normal cells. In contrast, BMI1 did not compensate for the negative effect of MK3 depletion on cell proliferation. MK3 overexpression inhibited the proliferation of normal and cancer cells. In contrast, MK3 depletion enhanced the cell cycling of cancer cell lines. Our data supports the idea that MK3-mediated abnormal M/SAPK signaling intersects with known checkpoints and contributes proliferative control. The potential implications of these observations for tumorigenesis are discussed.

## Results

### Gain or loss of MK3 function induces senescence in normal human fibroblasts

**Fig 1:**
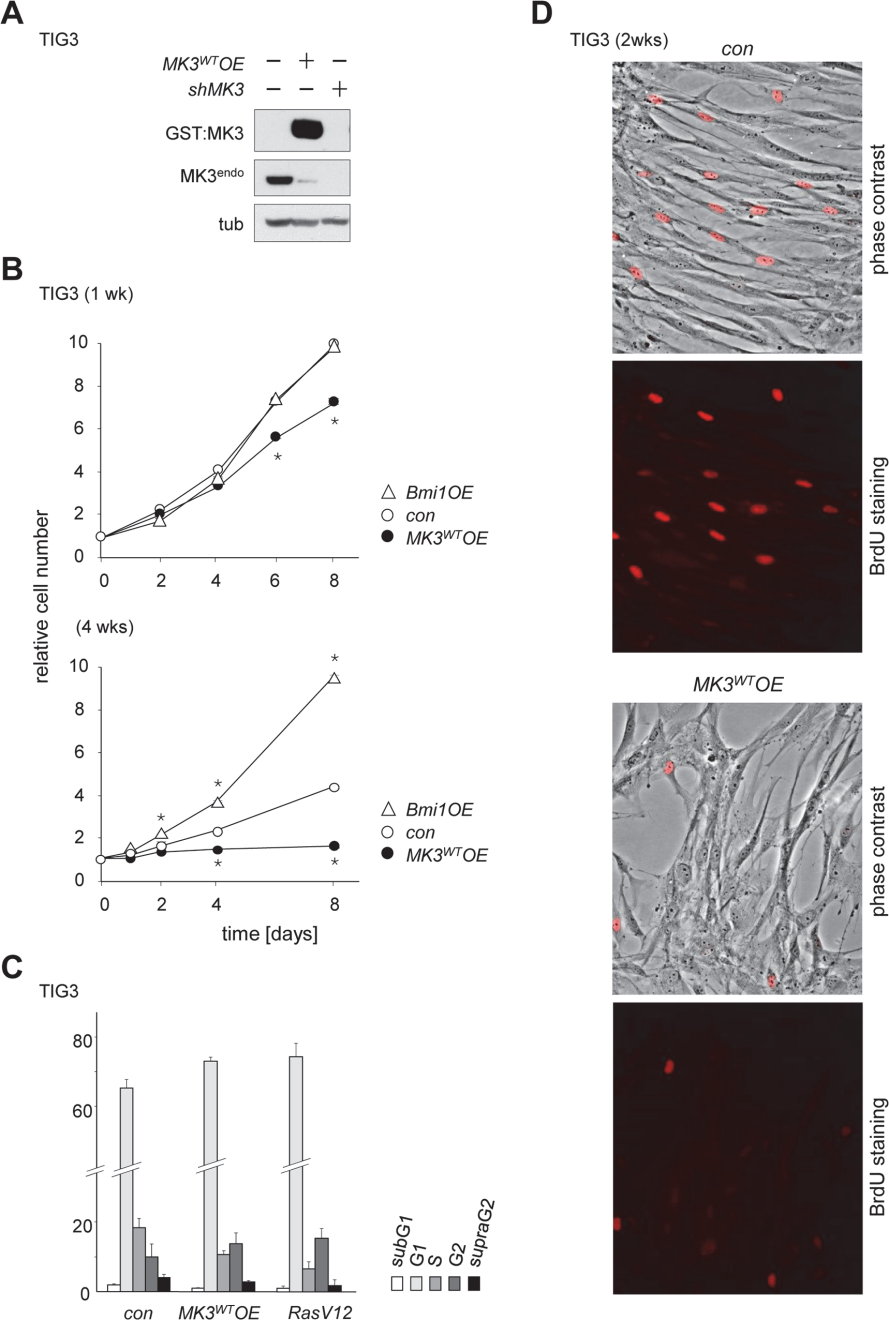
*MK3* ^*WT*^ overexpression induces a proliferative arrest in normal human fibroblasts. (A) Retroviral expression systems were applied to enhance (*MK3* ^*WT*^ *OE*) or remove (*shMK3*) MK3 expression. Western blot shows MK3 proteins: endogenous (MK3^endo^) and overexpressed (GST:MK3). (B) Proliferation curves of TIG3 cells transduced with: a retroviral *MK3* expression vector (*MK3* ^*WT*^ *OE*; filled circles), an empty vector (con; open circles) or a murine *Bmi1* expression vector (open triangles); proliferation was determined at 1 week (top panel) or ±4 weeks (bottom panel) after retroviral transduction of TIG3 cells. Cell counts at t = 2 through t = 8 were normalized to cell counts at t = 0 for each transduced cell culture individually (see Methods section for details); statistical significance was determined by two-tailed Student’s t-test and is presented relative to the empty vector control (* p < 0.05). (C) Quantification of DNA profiles (BrdU pulse-labeling and S-phase quantification by FACS) in TIG3/*MK3* ^*WT*^ *OE* cells at approximately 1 week post-transduction. *RasV12*-transduced cells were used as a positive control. (D) *MK3* ^*WT*^ *OE* reduces *de novo* DNA synthesis in TIG3/*MK3* ^*WT*^ *OE* cells; cell counts: control (con) 699 BrdU-positive cells/10 fields; *MK3* ^*WT*^ *OE*: 442 BrdU-positive cells/9 fields.

To study the role of MK3 in cellular processes related to cell proliferation, we modulated cellular MK3 levels in different cell models using retroviral expression systems and measured the effects thereof. We initially focused on normal diploid human TIG3 fibroblasts to determine the effect of forced overexpression of wild type (non-mutated) *MK3* (*MK3* ^*WT*^ *OE*) on cell proliferation (Fig 1A). Transduced TIG3 cells were expanded under selection pressure; proliferative capacity of these cultures was assessed at ±1 week and ±4 weeks after transduction. TIG3/*MK3* ^*WT*^ *OE* displayed clearly decreased proliferative capacity relative to empty vector control cells at the early time point and had altogether stopped dividing at 4 weeks post-transduction (Fig 1B). By means of reference, TIG3/*Bmi1OE* maintained proliferative capacity throughout the duration of the experiment, whereas primary empty vector-transduced TIG3 (*con*) progressively lost proliferative capacity due to limited proliferative lifespan *in vitro* (Fig 1B). DNA-profiling at late time points post-transduction revealed an increased number of cells in G0/G1 at the expense of cells in S-phase upon *MK3*-overexpression (Fig 1C); overexpression of the *RASV12* oncogene reduced S-phase cell numbers as anticipated as RASV12 is known to evoke oncogene-induced senescence (OIS) [16], Fig 1C). Reduced *de novo* DNA synthesis in TIG3/*MK3* ^*WT*^ *OE* cells was confirmed by a markedly lower number of BrdU-positive cells in TIG3/*MK3* ^*WT*^ *OE* cultures and (Figs 1D and S1A); equal relative distribution of cells throughout S-phase suggested that TIG3/*MK3* ^*WT*^ *OE* cultures had undergone an intraS-phase arrest, a feature known to occur in the context of oncogene-induced senescence (OIS) (S1B Fig).

**Fig 2:**
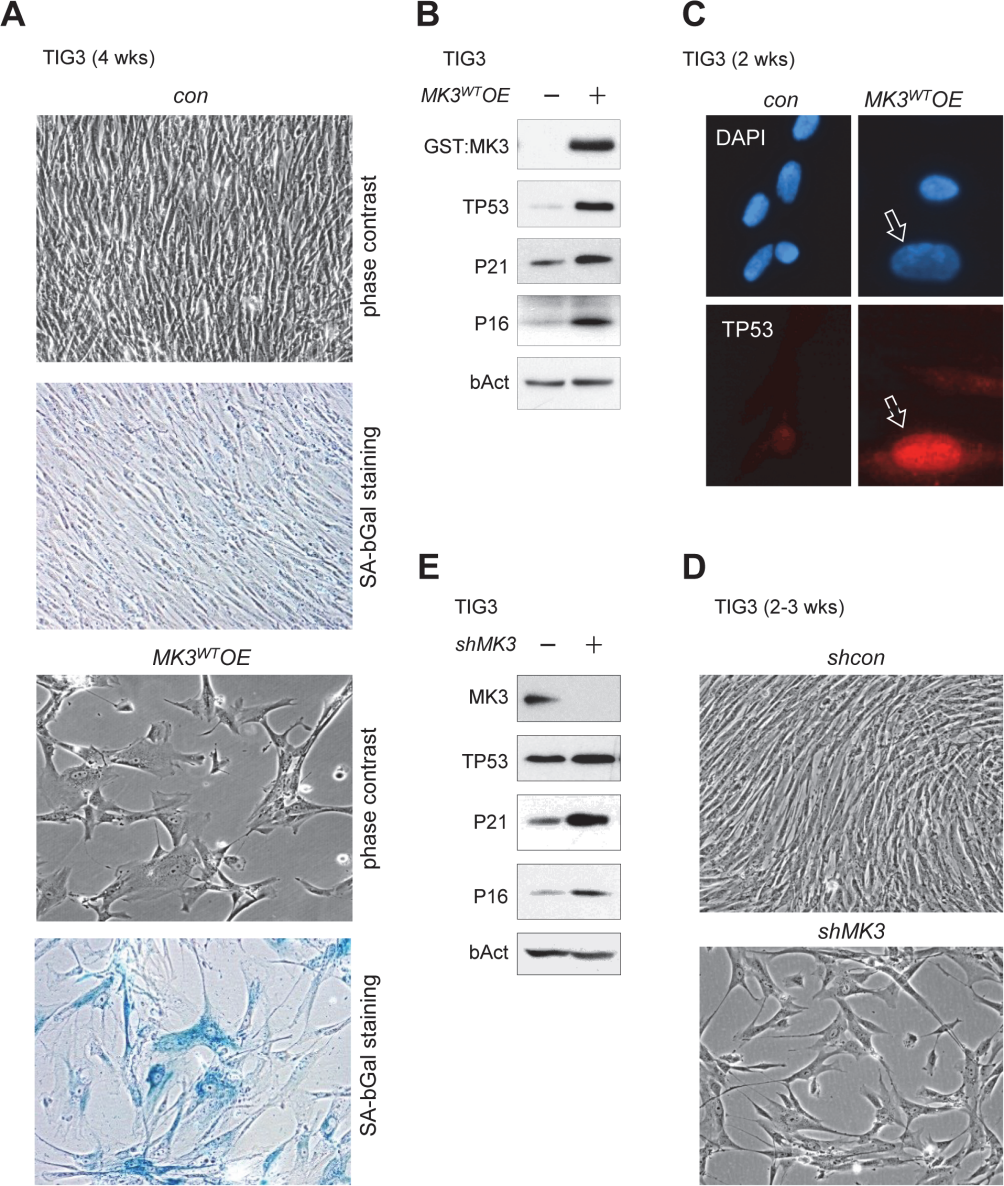
The *MK3* ^*WT*^ *OE*-induced proliferative block in normal cells correlates with induction of known senescence markers. (A) Cell morphology in TIG3/*MK3* ^*WT*^ *OE* cells (phase contrast; right upper panel); senescence-associated beta-Galactosidase (SA-bGal) staining in TIG3/*MK3* ^*WT*^ *OE* cells (right lower panel). (B) Protein expression levels of MK3, TP53, P21^CIP1/WAF1^ (P21) and P16^INK4A^ (P16) in TIG3/*MK3* ^*WT*^ *OE* (*MK3* ^*WT*^ *OE*) *versus* control (*con*) cell lysates; loading control: b-Actin (bAct). TIG3/*MK3* ^*WT*^ *OE* cells were cultured for approximately 3–4 weeks prior to extraction. (C) Nuclear TP53 accumulation in senescent TIG3/*MK3* ^*WT*^ *OE* cells; counterstaining: DAPI. (D) Phase contrast images showing cello morphology in TIG3/*shMK3* cells (*shMK3*, lower panel; phase contrast) *versus* control cells (*shcon;* upper panel). Retrovirally transduced TIG3 cells (*shcon* and *shMK3*) were submitted to puromycin selection for 3–4 days, and plated at equal density (20–25% confluency) at the onset of the experiment; pictures were taken when *shcon* cells reached confluency. (E) Analysis of protein expression in TIG3/*shMK3* and control (*shcon*) cell lysates: TP53, P21^CIP1/WAF1^ (P21) and P16^INK4A^ (P16); loading control: b-Actin (bAct).

In further support of activation of a senescence-associated response by MK3, TIG3/*MK3* ^*WT*^ *OE* cells displayed enlarged, flat-cell morphology (Fig 2A). The occurrence of senescence was further corroborated by expression of the senescence-associated beta-Galactosidase (SA-bGal) marker protein in large flat cells (Fig 2A). *MK3* ^*WT*^-overexpression also negatively affected cell division in immortal TIG3^*hTERT*^ cells, indicating that MK3 acts downstream or independent of hTERT in proliferative control (S1C Fig). TP53 is a senescence marker, activated downstream of e.g. increased mitogenic signaling through RAS/ERK [17–20]. Consistent with the observed *MK3*-mediated senescence response, global expression of TP53 was elevated in TIG3/*MK3* ^*WT*^ *OE* cells (Fig 2B). Increased staining of TP53 was readily detectable in senescent TIG3/*MK3* ^*WT*^ *OE* nuclei (Fig 2C); likewise expression of P21^CIP1/WAF1^, a direct transcriptional target of TP53, was increased (Fig 2B). The increased expression of *P16* ^*INK4a*^ in relation to sustained *MK3* ^*WT*^ *OE* was consistent with its crucial role in establishing irreversible senescence (Fig 2B) [17,21,22]. We next tested whether the adverse effect of *MK3* ^*WT*^ *OE* was dependent on its kinase activity. Surprisingly, the early effect of *MK3* ^*WT*^ *OE* on cell proliferation occurred independent of MK3 kinase activity, as expression of a kinase-dead mutant (*MK3* ^*KM*^ *OE*) and a constitutively active mutant (*MK3* ^*CA*^ *OE*) did not affect cell proliferation at 1 week post-transduction (S2A Fig). In agreement with this, cellular TP53 levels were only moderately increased in the TIG3/*MK3OE* cells, not in TIG3/*MK3* ^*KM*^ *OE* or TIG3/*MK3* ^*CA*^ *OE* cells. P16^INK4A^ levels were substantially increased at 1 week post-transduction in both TIG3/*MK3* ^*WT*^ *OE* and TIG3/*MK3* ^*CA*^ *OE* cultures, despite the differences in proliferation rate (S2A and S2B Fig). P16^INK4A^ was also induced by *MK3* ^*KM*^ *OE*, however, at a lower level than in both other conditions. Despite the initial absence of adverse effects on proliferation, all three MK3 variants reduced proliferation rate at 4 weeks post-transduction (S2A and S2B Fig). The absence of correlation between TP53 and P16^INK4A^ levels and proliferation rate at 4 weeks suggests a potential contribution of additional molecular targets and mechanisms to the observed effects on proliferation. Combined this data shows that overexpression of MK3^WT^ or two MK3 kinase mutants in human diploid fibroblasts reduces their proliferative capacity.

As the *MK3* locus is frequently missing in cancers as part of often larger genomic deletions [5,6], it is conceivable that cellular MK3 depletion affects cell cycle regulation and contributes to tumorigenesis. To study the effect of loss of MK3 on cell proliferation, we next reduced cellular MK3 levels using RNA-interference. To this end a retroviral *shRNA* construct was designed that selectively targeted *MK3* (*shMK3*) (S2C Fig; *cf*. Fig 1A). Similar to *MK3* overexpression in TIG3 cells, MK3 depletion resulted in reduced proliferation of TIG3/*shMK3* cells and increased flat cell formation (Fig 2D), which was confirmed by reduced BrdU-incorporation in TIG3/*shMK3* cultures (S2D Fig). The *shMK3*-associated arrest in normal cells was accompanied by increased TP53, P21^CIP1/WAF1^ and P16^INK4A^ expression (Fig 2E). As MK2 is also expressed in TIG3 cells (*cf*. S5B Fig) we aimed to exclude the possibility that MK2 confounded the observed MK3 depletion-associated proliferative phenotype. To this end TIG3 cells were either transduced with a *shMK3* or a *shMK2/3* construct; the latter effectively reduced expression of both MK2 and MK3 (S2C Fig). Both constructs increased G1-arrest with equal efficiency; MK2 neither compensated for loss of MK3 nor added to the effect of MK3 depletion, suggesting that the observed proliferative reduction could be attributed to MK3 in this experimental setting (S2E Fig). Thus, overexpression as well as depletion of MK3 in TIG3 cells induced a proliferative block, which correlated with expression of known senescence markers. Taken together, these results suggested that MK3 controls proliferative lifespan in normal diploid human fibroblasts.

### Genetic interaction between MK3 and Polycomb in proliferative lifespan

**Fig 3:**
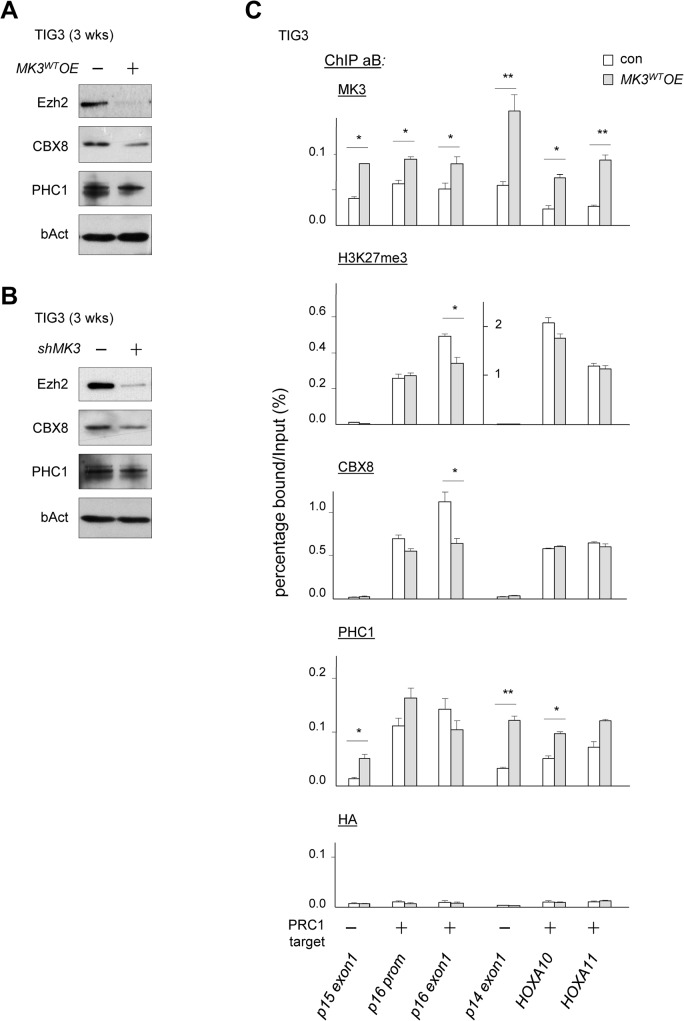
Functional links between MK3 and PRC in proliferative life span. (A) Expression levels of the PRC proteins EZH2, CBX8 and PHC1 in senescing TIG3/*MK3* ^*WT*^ *OE* cells at ±3–4 weeks post-transduction; loading control b-Actin (bAct). (B) Expression levels of the PRC proteins EZH2, CBX8 and PHC1 in senescing TIG3/*shMK3* cells at ±2–3 weeks post-transduction; loading control b-Actin (bAct). (C) Chromatin immunoprecipitation (ChIP) analysis of MK3, H3K27me3, CBX8, PHC1 enrichment in *MK3* ^*WT*^ *OE* (*MK3* ^*WT*^ *OE*) expressing and control (*con*) TIG3 cells; PRC1-target loci (p*16 promoter*, *p16exon1*, *HOXA10*, *HOXA11)* and non-target loci (p15exon1, p14exon1) are indicated below the figure. Enrichments are presented as percentages of total input. Negative control HA antiserum. Experiments were performed three times; results of one representative experiment are shown (statistical significance: * p<0.05, ** p<0.01; t-test).

MK3 has been identified as a PRC1 binding partner; we have shown that signaling through ERK1/2, P38 and MK3 controls gene expression via modulating chromatin-association of the PRC1 complex [8,14]. We further explored the functional interaction between PRC-function and MK3 in the context of cell proliferation. Senescent cells are known to release *CDKN2A/INK4A* repression and show increased P16^INK4A^ and/or P14^ARF^ levels [22–24]. Increased expression at the *CDKN2A* locus correlates with reduced local H3K27me3 [25,26]. Expression of the responsible histone H3K27 methyltransferase (HMT), the PRC2-class protein EZH2, was reported to be progressively reduced by TP53 in senescing cells [27,28]. Transcriptional repression of the *CDKN2A*-locus in proliferating cells correlates with PRC2-mediated histone H3 Lysine 27 trimethylation (H3K27me3) [25,26]. In good agreement with these reports, we observed that global TP53 and EZH2 protein levels inversely correlated in senescent TIG3/*MK3* ^*WT*^ *OE* cultures and that reduced cellular EZH2 correlated with increased *P16* ^*INK4A*^ expression (Fig 3A; *cf*. Fig 2B). Reduced EZH2 levels also inversely correlated with *P16* ^*INK4A*^ expression in senescent TIG/*shMK3* cultures (Fig 3B), in further support of a functional link between EZH2 reduction and senescence upon modulation of MK3 levels. In addition to EZH2, CBX8 levels were reduced in both senescent TIG3/*MK3* ^*WT*^ *OE* and TIG3/*shMK3* cultures, whereas PHC1 levels appeared relatively unaffected by gain or loss of MK3 function (Fig 3A and 3B). Thus far, the combined data shows that MK3 modulation induces a state of senescence that correlates with reduced cellular EZH2 levels and elevated *P16* ^*INK4A*^ expression.

To assess changes in PRC1 protein/chromatin-association and Polycomb-related status at the *CDNK2A/INK4A* locus in relation to MK3 overexpression, we applied chromatin immunoprecipitation (ChIP) technology to pre-senescent TIG3/*MK3* ^*WT*^ *OE* cell cultures. The *CDKN2A/INK4A* locus has been mapped before in detail in regards to H3K27me3-marking and PRC1-occupation in proliferating and senescent cells [25,26]. A number of previously identified PRC1-negative (*p15exon1*; *p14exon1*) and positive (*HOXA10*; *HOXA11*) regions were used as controls in our analyses [25]. MK3-overexpression moderately, but consistently enhanced chromatin-occupation of MK3 at all loci probed including non-PRC1 target loci (Fig 3C), in line with our earlier observation that part of cellular MK3 associates with chromatin [8]. Absence or presence of H3K27me3-marking in our experimental model correlated perfectly with absence or presence of CBX8 occupation, respectively (Fig 3C); CBX8 binds the K27 trimethyl-mark through its chromobox domain [25,29]. Interestingly, chromatin-occupation of PHC1, a direct binding partner of MK3 [8], increased in parallel with MK3 at all promoter sequences analyzed, including at non-PRC1 target loci (Fig 3C). In contrast to CBX8, PHC1 was found associated also with non-PRC1 target loci, suggesting that (part of) PHC1 may associate with chromatin in an H3K27me3-indendent fashion (Fig 3C). Relevantly, MK3-associated induction of senescence correlated with loss of H3K27me3-enrichment at *p16-exon1* and CBX8-displacement from the *P16* ^*INK4A*^ locus (promoter and exon1; Fig 3C). This data is in good agreement with both the reduced cellular EZH2 and CBX8 levels, and with the increased *P16* ^*INK4A*^ expression (S3B Fig) [25,26]. Combined, this data shows that in the MK3^WT^-overexpressed state, cellular senescence correlates with reduced cellular EZH2 and CBX8 levels and consequential de-repression of transcription at the *CDKN2A* locus.

**Fig 4:**
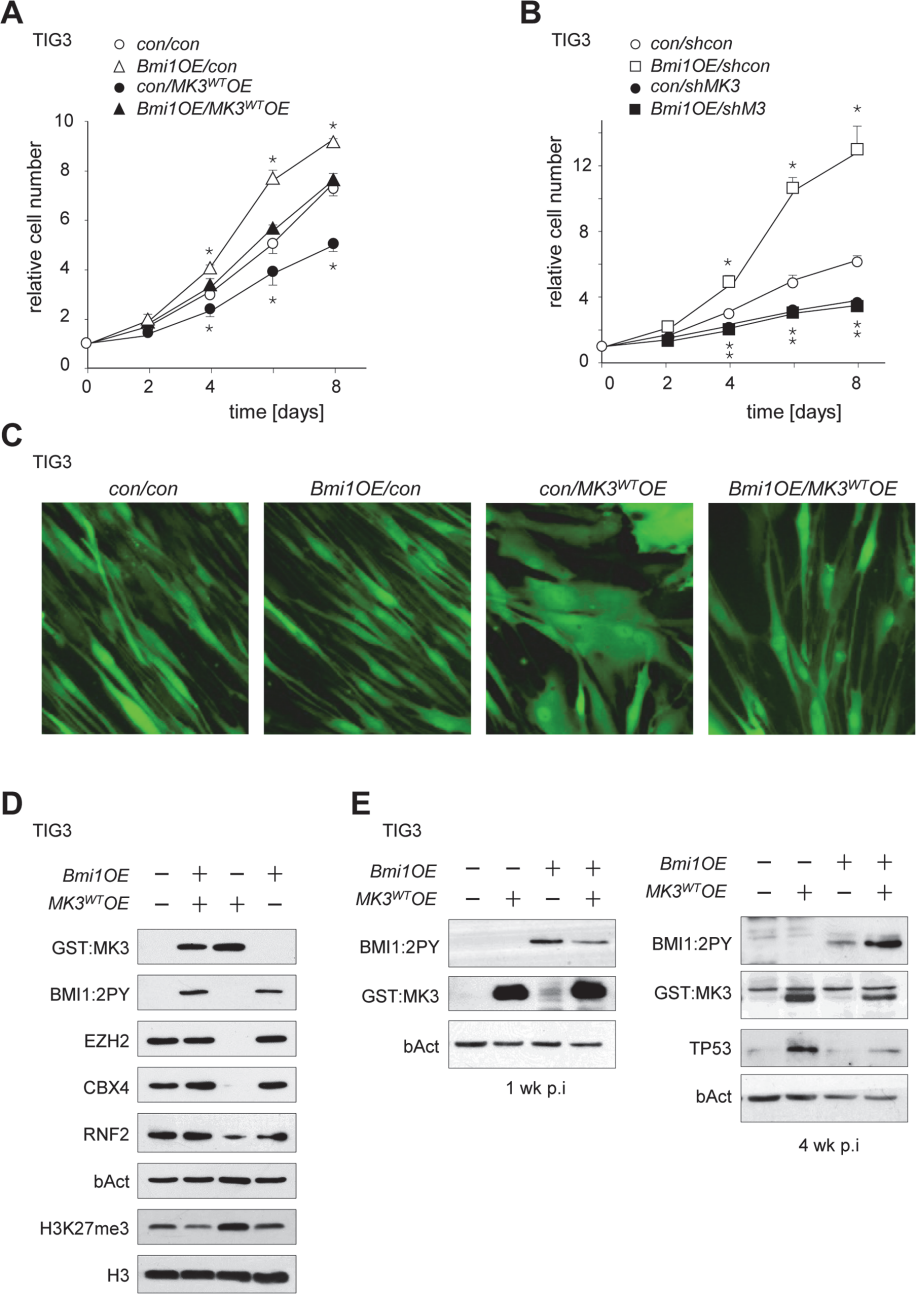
Functional links between MK3 and PRC in proliferative life span. TIG3 cells were sequentially transduced with either *Bmi1*.*ires*.*GFP* (*Bmi1OE*) or *GFP* (con) virus, and *MK3*/*puromycin* (*MK3OE*) or control *puromycin* virus (con) at 48 hrs intervals. Retroviral vectors expressing murine *Bmi1/*GFP reporter were transduced first (or empty vector control), followed by a MK3/*puromycin* resistance marker (or empty vector control). Transduction of *Bmi1OE* and control transduced cells was simultaneously carried out with the same *MK3* ^*WT*^ *OE* viral preparation (or control virus) to minimize inter-experimental variation. Cells were grown on selection medium and proliferation capacity was tested ± 2–3 weeks post-transduction. (A) Proliferation curves of normal human TIG3 fibroblasts transduced with a retroviral *MK3* ^*WT*^ *overexpression* vector (*MK3* ^*WT*^ *OE*; black symbols) or *shcon* vector (white symbols), in conjunction with either an empty vector control (con; circles) or a murine *Bmi1* expression vector (*Bmi1OE*; triangles). (B) Proliferation curves of normal human TIG3 fibroblasts transduced with a retroviral *MK3* knock-down vector (*shMK3*; black symbols) or *shcon* vector (white symbols), in conjunction with either an empty vector control (con; circles) or a murine *Bmi1* expression vector (*Bmi1OE*; squares). Cell counts at t = 2 through t = 8 (A, B) were normalized to cell counts at t = 0 for each transduced cell culture individually (see Methods section for details); statistical significance was determined by two-tailed Student’s t-test and is presented relative to the empty vector control (* p < 0.05). (C) Comparative morphology of TIG3 cells expression *Bmi1* and/or *MK3* versus control cells as recorded by GFP fluorescent imaging ± 3 weeks after transduction (D) Immunoblot analysis of EZH2, CBX4, RNF2 and H3K27me3 in *MK3* ^*WT*^ *OE*, *Bmi1OE*, *Bmi1OE/MK3* ^*WT*^ *OE* and *control* TIG3 cell lysates. (E) Expression analysis of BMI1, MK3, and TP53 at the indicated time points in (corresponding to experiment in Fig 4C). Cells were grown on selection medium and analysed at 1 or 4 weeks after serial transduction; expression vectors and antibodies are indicated in the figure. Early and late samples were loaded on the same gel for BMI1 analysis; corresponding sections are shown separately.

We next explored the functional relationship between MK3 and BMI1 in proliferative lifespan. Genetic ablation of PRC1-members, among which BMI1 and PHC paralogs, is known to induce premature cellular senescence *in vitro* and *in vivo* [30,31]. In the light of the herein described local (*i*.*e*. at the *CDKN2A* locus) chromatin-displacement of PRC1 in senescing cells and our previous observation that prolonged *MK3* overexpression leads to reduction of chromatin–associated BMI1 [8], we speculated that *Bmi1OE* would overcome the senescent state induced by gain or loss of MK3 function. To determine whether *Bmi1OE* could bypass the negative effect of *MK3OE* on proliferative capacity, TIG3 fibroblasts were sequentially transduced with murine *Bmi1* and *MK3-*encoding retroviral expression vectors. *Bmi1OE* consistently produced a proliferative advantage for TIG3 fibroblasts (TIG3/*Bmi1OE/MK3* ^*WT*^ *OE*) (Fig 4A and 4B). Whereas *MK3* ^*WT*^ *over*expression alone reproducibly showed reduced TIG3 proliferation, co-expression of *Bmi1* compensated for the adverse effects of *MK3* ^*WT*^ overexpression on cell proliferation (Fig 4A). Importantly, in contrast to MK3^WT^ expression, the adverse effect of MK3 ablation on fibroblast proliferation was not rescued by BMI1 (Fig 4B). *Bmi1 co-*expression also reversed the MK3-induced senescent cell morphology in observed in TIG3/*MK3* ^*WT*^ *OE* cultures, (Fig 4C). Co-expression of *Bmi1* in TIG3/*MK3* ^*WT*^ *OE* cells prevented loss of EZH2 as well as of the PRC1 complex proteins CBX4 and RNF2 (Fig 4D). Finally, the global increase of H3K27me3 observed in TIG3/*MK3* ^*WT*^ *OE* cells was prevented by co-expression of Bmi1 (Fig 4D). TP53 expression in TIG3/*MK3* ^*WT*^ *OE* cells was countered by BMI1 co-expression (Fig 4E). Relevantly, sequentially transduced fibroblast cultures co-expressing *Bmi1/MK3* ^*WT*^ self-selected for increased BMI1 protein levels over time compared to cultures overexpressing *Bmi1* alone (Fig 4E), further supporting of a genetic interaction between MK3 and BMI1 in the context of cell proliferation. As EZH2 targets the *CDKN2A/INK4A* locus for transcriptional repression, we also examined whether EZH2 alone could rescue the *MK3*-induced proliferative arrest. Whereas *Bmi1* co-expression in TIG3 cells clearly reduced *MK3* ^*WT*^ *OE*-induced P16^INK4^ expression in the same experiment, *EZH2OE* did not prevent MK3^WT^-induced upregulation of cellular P16^INK4^; instead, elevated basal P16^INK4^ expression both at the mRNA and protein levels was already detectable in control cells in TIG3/*EZH2OE* cells (S4A and S4B Fig). *Bmi1* co-expression clearly neutralized the adverse effects of *MK3* ^*WT*^ *OE* on cell proliferation, the effect of *EZH2OE* on the *MK3* ^*WT*^ *OE*-proliferation phenotype were inconsistent, yet never compensatory (data not shown). Taken together, the above findings support a functional genetic interaction between MK3 and BMI1/PRC1 in proliferative control of normal human fibroblasts, and suggest that EZH2/PRC2 is likely neither sufficient to bypass MK3-arrested proliferation nor to prolong proliferative lifespan.

### Loss of MK3 provides a growth advantage in cancer cell models

We used the large-scale cancer genome-sequencing and expression analyses initiative *Cancer Cell Line Encyclopedia* (CCLE; http://www.broadinstitute.org/ccle) to assess MK3 expression in nearly 1000 cancer cell lines [32]. MK3 was relatively high expressed in various cancer cell types, among which bone, pancreatic, colorectal and endometrial cancers (S5A Fig). Conversely, MK3 showed significant focal deletion across a set of more than 3000 tumors, or its expression is low/absent in cancer cell types, including small-cell lung cancers, neuro- and medulloblastomas [32]. Although cancers of (neur)ectodermal origin tend to show low/absent MK3 expression, there was no obvious correlation between germ-layer origin and MK3 expression among the various cancer types (S5A Fig), suggesting that MK3 status is an acquired feature in tumors. When we next examined MK3 protein levels in a panel of cancer cell lines, we were able to confirm large variation in MK3 expression between different cell lines (S5B Fig); no obvious correlation was observed between MK3 and MK2 levels, in line with their distinct cellular functions [33,34]. Combined, this data points to a possible involvement of MK3 in tumorigenesis and suggests that the role of MK3 may be determined by cellular and/or genetic context.

**Fig 5:**
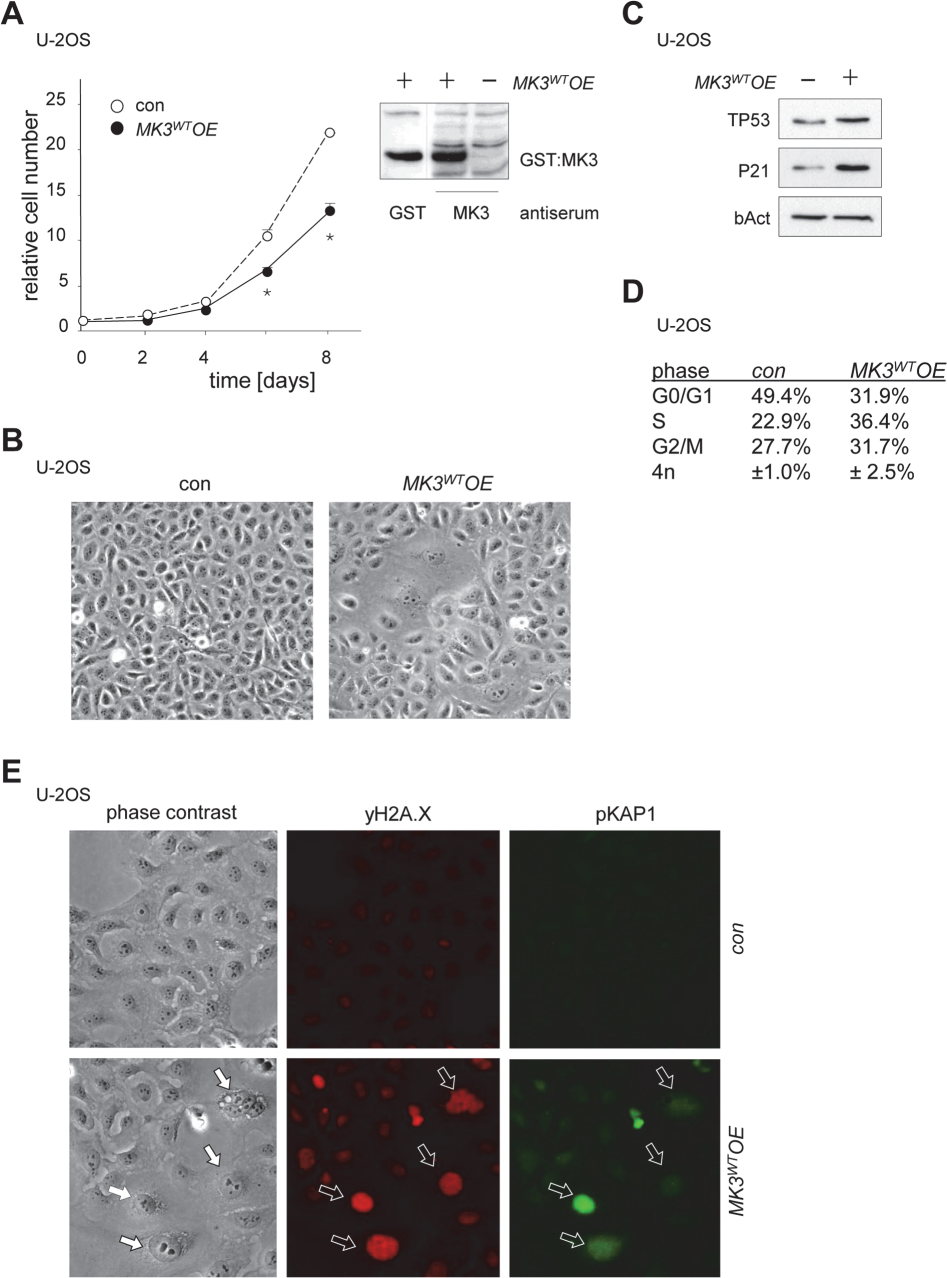
Proliferative regulation by MK3 in cancer cell lines. (A) Proliferation curves (left) of U-2OS cells expressing a retroviral *MK3* vector (*MK3* ^*WT*^ *OE*; filled circles) or an empty vector (con; open circles); overexpression of GST-MK3 (*MK3* ^*WT*^ *OE*) in U-2OS cells detected with and GST or a MK3-antiserum (right panel). Cell counts at t = 2 through t = 8 were normalized to cell counts at t = 0 for each transduced cell culture individually (see Methods section for details); statistical significance was determined by two-tailed Student’s t-test and is presented relative to the empty vector control (* p < 0.05). (B) Phase contrast images showing cell morphology in U-2OS/*MK3* ^*WT*^ *OE* cells and control cells. (C) Protein expression levels of the check-point regulator proteins TP53 and p21^CIP1/WAF1^ (P21) in U-2OS/*MK3* ^*WT*^ *OE* cells; loading control b-Actin (bAct). (D) DNA profile analysis of U-2OS/*MK3OE* versus control cells (4–6 days post-transduction; representative experiment). *MK3* ^*WT*^overexpression elicits an intra S-phase arrest: table shows a substantially increased S-phase occupancy. (E) Immunohistochemical staining for phosphorylated H2A.X (γH2A.X) and phosphorylated KAP1pSer824 (pKAP1; arrows) to visualize DNA damage in U-2OS/*MK3* ^*WT*^ *OE* cultures; control (top panels) or *MK3* ^*WT*^ *OE* (bottom panels).

Given its possible dual involvement in tumorigenesis, we probed cancer cell lines for effects of gain or loss of MK3 function on cell proliferation. We here focused on U-2OS osteosarcoma cells and the HeLa cervical carcinoma cells. Of note, U-2OS/*MK3* ^*WT*^ *OE* cultures also showed an *MK3* ^*WT*^ *OE*-induced reduction of proliferation and enhanced flat cell morphology (9.6% ±1.8 *MK3* ^*WT*^ *OE vs*. 2.3% ±0.8 con, *P*<0.001) and expression of SA-bGal (Fig 5A, 5B and S6A Fig). HeLa/*MK3OE* cells also showed enhanced flat cell induction (*data not shown*). Consistent with a response resembling senescence, U-2OS/*MK3* ^*WT*^ *OE* cultures showed enhanced expression of TP53 and of its transcriptional target P21^CIP1/WAF1^ (Fig 5C). Consistent with the involvement of the TP53 pathway in DNA damage response (DDR), nuclei of flat U-2OS/*MK3* ^*WT*^ *OE* cells showed increased co-staining for TP53, P14^ARF^ and P21^CIP1/WAF1^ (S6B Fig). Increased replication stress is known to induce a proliferative arrest which is often associated with double strand DNA breaks (DSB). The occurrence of replication stress-associated DSB is typically associated with an initial intraS-phase arrest, as part of a DDR [19,35,36]. U-2OS/*MK3* ^*WT*^ *OE* cells showed a ±1.5 fold increase of cells in S-phase within days after transduction (Fig 5D). To determine whether the flat cell phenotype in U-2OS/*MK3OE* cells was associated with DNA damage, we use immunofluorescence to study the occurrence of DSB by measuring phosphorylation of histone variant H2A.X (γH2A.X) and of KAP1 phosphorylation at serine 824 (pKAP1). Double-positive γH2A.X/pKAP1 cells were found in considerable numbers throughout U-2OS/*MK3* ^*WT*^ *OE* cultures; the nuclei of enlarged flat cells were prominently stained (Fig 5E).

**Fig 6:**
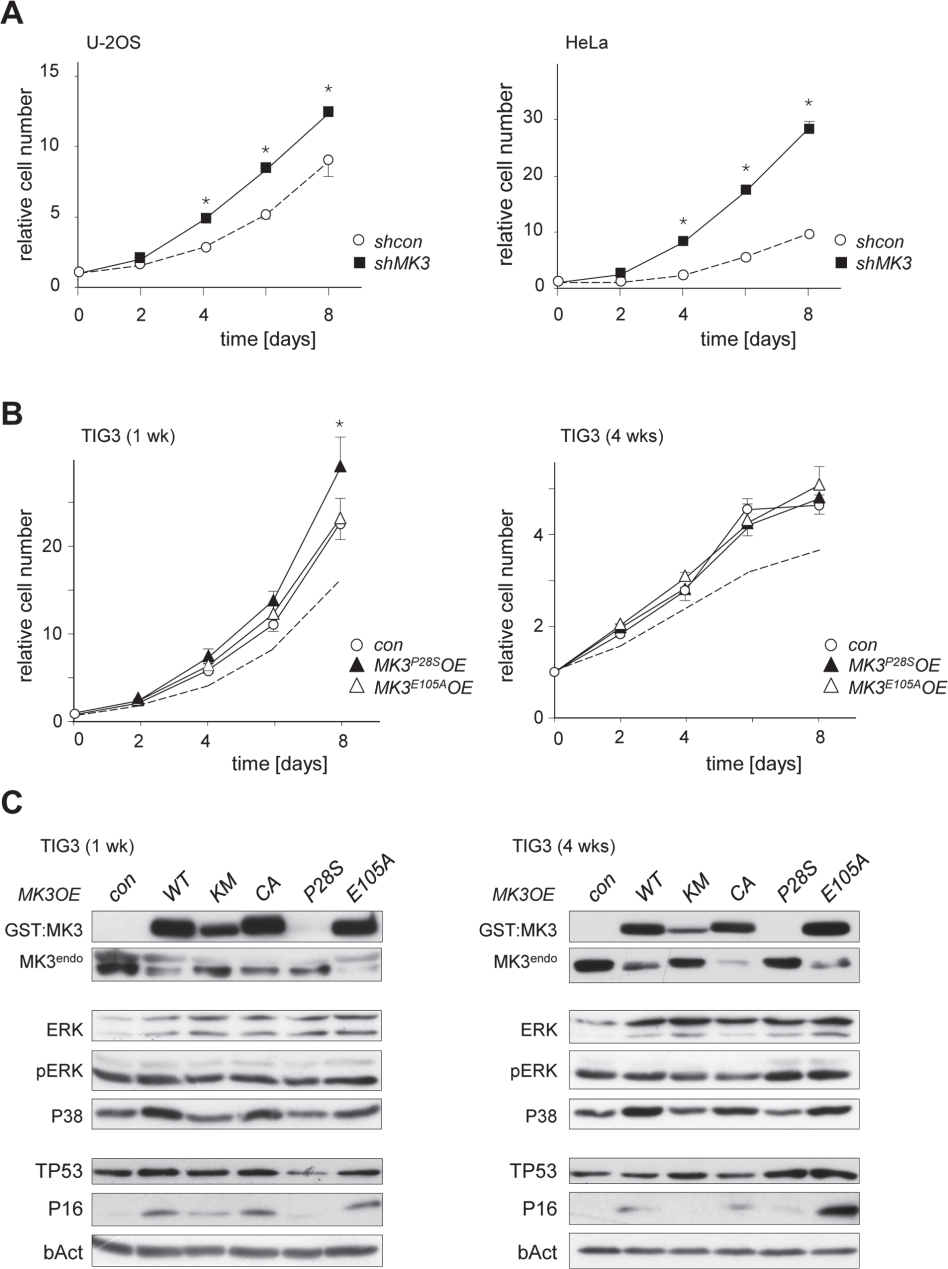
Modulation of MK3-levels leads to M/SAPK signalling imbalance. (A) Proliferation profiles of U-2OS/*shMK3* cells (left panel; filled squares)) and HeLa/*shMK3* cells (right panel; filled squares) *versus* control (*shcon*; open circles). (B) Proliferation profiles of TIG3 cells expressing MK3 mutants MK3P28S (*MK3* ^*P28S*^ *OE*; filled triangles) or MK3E105A (*MK3* ^*E105A*^ *OE*; open triangles) at 1 week post-transduction (left panel) and 4 weeks post-transduction (right panel). The dotted lines in the graphs represent the MK3WT proliferation profiles, as depicted in S2A Fig. Cell counts at t = 2 through t = 8 (A, B) were normalized to cell counts at t = 0 for each transduced cell culture individually (see Methods section for details); statistical significance was determined by two-tailed Student’s t-test and is presented relative to the empty vector control (* p < 0.05). (C) Protein expression profiles of GST-tagged MK3 (GST:MK3), endogenous MK3 (MK3^endo^), ERK, phosphorylated ERK (pERK) P38, TP53 and p16^INK4A^ (P16) in TIG3 cells at indicated timepoints post-transduction (corresponding to Fig 4B); loading controls: b-Actin (bAct).

As the parental U-2OS and HeLa cell lines both express moderate levels of MK3 (*cf*. S5B Fig), we studied the effect of RNAi-mediated MK3 depletion on cell proliferation. Remarkably, both U-2OS and HeLa cells proliferated faster in the absence of MK3 (Fig 6A). Thus, in contrast to the negative effects of gain and loss of MK3 function on proliferative capacity in in normal human fibroblasts, MK3^WT^ overexpression and ablation produce opposing effects in cancer cell models.

### MK3 modulation or mutation induce signaling imbalance

The finding that proliferation capacity of U-2OS and HeLa cells was decreased in the context of *MK3OE* was unexpected. U-2OS osteosarcoma and HeLa cervical carcinoma cells both retain a “wild type” genetic status for *TP53* and *pRB* and both carry a genetically intact *CDKN2A/INK4A* locus [37,38]. Both TP53 and pRB-dependent checkpoints are, however, compromised in these cells (see Discussion section). In U-2OS cells, the *P16/INK4A* promoter is transcriptionally silenced due to CpG methylation [38,39]. HeLa cells have an intrinsically high P16^INK4A^ level; the pRB/P16^INK4A^ checkpoint is dysfunctional in these cells, however, as pRB is continuously targeted for proteolytic degradation by HPV-expressed oncoprotein. Combined with our earlier observation that changes in proliferation rate of TIG3/*MK3* kinase mutant cultures did not correspond well with TP53 and/or P16^INK4A^ levels, this prompted us to search for involvement of additional molecular mechanisms that may contribute to reduced proliferation in the context of gain or loss of MK3 function. MK3 is a downstream target of the M/SAPKs P38, JNK as well as ERK [4]; hence, MK3 acts as a convergence point of mitogenic and stress signaling cascades. We previously showed that gain, loss or inhibition of MK3 function affected expression levels of P38 and JNK and altered phosphorylation dynamics of ERK, P38 and JNK in response to mitogenic stimulation in U-2OS cultures [14]. As these findings pointed to an MK3-dependent M/SAPK signaling imbalance, we aimed to determine the effects of MK3 modulation in the context of cell proliferation of normal human fibroblasts and in cancer cell models. Whereas ERK and pERK (phosphorylated ERK) levels were only marginally affected under standard culturing conditions in TIG3/*MK3* ^*WT*^ *OE* cells, JNK, P38 and phosphorylated P38 (pP38) levels were substantially increased in *MK3* ^*WT*^ *OE* fibroblasts (S7A Fig). P38 expression was sustained during prolonged culturing (S7B Fig). P38 levels were increased in *MK3* ^*WT*^ *OE* U-2OS and HeLa cultures (S7C Fig), suggesting a potential common involvement of P38 in the *MK3* ^*WT*^ *OE*-associated proliferation effects in normal and cancer cells. Conversely, yet in keeping with the increased P38 levels in *MK3OE* cultures, P38 levels were reduced in response to MK3-depletion (*shMK3*) in both U-2OS and HeLa cancer cell lines (S7D Fig). In light of our previous finding that MK3 serves to provide negative regulatory feedback on canonical MEK/ERK signaling [14], and the current observation that P38 and JNK levels are increased in *MK3* ^*WT*^ *OE* cells, we speculated that a dysbalance in MAPK and SAPK activity could contribute to the observed effects of MK3 modulation on cell proliferation in the various cell models. In keeping with this notion, the proliferation rate of U-2OS/*MK3* ^*WT*^ *OE* cells was negatively affected by reduction of serum concentration in a dose-dependent manner and serum deprivation (1% FCS) significantly enhanced flat cell formation in U-2OS/*MK3* ^*WT*^ *OE* cultures (S7E Fig; *cf*. Fig 5B). This data supports the idea that modulation of MK3 has direct functional consequences for mitogenic responses and supports a role for signaling imbalance caused by modulation of cellular MK3 levels in normal cells as well as in cancer cells.

A recent systematic cancer genome re-sequencing effort predicted potential oncogenic driver mutations in known and novel genes [7]. Among these potentially tumorigenic MK3 variants were a number of MK3 missense mutants, among which P28S and E105A. As the effect of these mutations on MK3 function is currently unknown, we used the TIG3 model to evaluate their effect on cell proliferation, P38 and ERK levels and P38 and ERK phosphorylation. Comparable to the MK3^KM^ and MK3^CA^ mutant constructs, neither MK3^P28S^ nor MK3^E105A^ had an attenuating effect on cell proliferation at 1 week post-transduction (Fig 6B); the proliferation rate of TIG3/*MK3* ^*P28S*^ *OE* was modestly but significantly increased over the (empty vector) control and TIG3/*MK3* ^*E105A*^. In contrast, at 4 weeks post-transduction none of the *in vivo* identified MK3 mutants mimicked the negative effect of the experimental kinase mutants (MK3^KM^, MK3^CA^) and the non-mutated MK3^WT^ kinase on proliferation (Fig 6B). Although all MK3 variants showed modestly enhanced total ERK1/2 levels, interestingly higher relative proliferation rate correlated with enhanced ERK levels (Fig 6C) in both MK3^P28S^ and MK3^E105A^ mutants at the 4 week time point. Under standard culturing conditions, TIG3/*MK3* ^*WT*^ *OE*, TIG3/*MK3* ^*CA*^ *OE* and TIG3/*MK3* ^*E105A*^ *OE* showed an sustained, increased P38 level, whereas this effect was less clear in the TIG3/*MK3* ^*KM*^ *OE* and TIG3/*MK3* ^*P28S*^ *OE*; the total P38 level in the latter cells seemed slightly lower at 4 weeks post-transduction (Fig 6C). In line with the earlier noted lack of correlation between proliferation characteristics and TP53 and/or P16^INK4A^ levels, TP53 levels were highest at 4 weeks post-transduction in the cells that had the highest relative proliferation rate. Similarly, absolute P16^INK4A^ levels were highest in TIG3/*MK3* ^*E105A*^OE cultures (Fig 6C). We previously established that gain, loss or inhibition of MK3 function resulted in abnormal mitogenic signalling as a result of altered phosphorylation dynamics of MEK/ERK [14]. We here show that mitogenic stimulation (*i*.*e*. serum starvation/serum stimulation), P38 levels and phosphorylation dynamics appeared dependent on the MK3 variant expressed. P38 levels were elevated in all TIG3/*MK3* ^*WT*^ *OE* cultures, irrespective of MK3 type, which is suggestive of a MK3-kinase activity independent effect (S8A and S8B Fig). TIG3/*MK3* ^*WT*^ *OE*, TIG3/*MK3* ^*CA*^ *OE* and TIG3/*MK3* ^*E105A*^ *OE* showed more intense P38 phosphorylation compared to TIG3/*MK* ^*KM*^ *OE* and TIG3/*MK3* ^*P28S*^ *OE*; in addition, pP38 levels were still high in the former three cultures at 2 hours post-stimulation, compared to TIG3/*MK* ^*KM*^OE and TIG3/*MK3* ^*P28S*^OE (S8B Fig). Taken together the above data provides the first evidence that MK3-mutation affects cell proliferation. TIG3 fibroblasts thus provide a useful model to evaluate potential tumorigenic effects of novel MK3 mutants on cell proliferation. Our findings suggest that *MK3* ^*P28S*^ and *MK3* ^*E105A*^ mutants exert specific effects on M/SAPK signalling. In addition, the data support the notion that M/SAPK signalling intersects with TP53 and pRB mediated cell cycle checkpoints.

## Discussion

The relevance of signaling through MK3 for cell proliferation was unknown. We here report that sustained gain or loss of MK3 induces a senescent state in normal human fibroblasts. Importantly, the MK3 overexpression-induced senescence is bypassed by co-expression of the oncoprotein BMI1, whereas the replicative senescent-like arrest induced by MK3 ablation is not. Surprisingly, *MK3* ^*WT*^ *OE* overexpression also induces a significant reduction of proliferation in cancer cells, whereas loss of MK3 enhances cancer cell proliferation. In conjunction with our previous finding that modulation of cellular MK3 levels or inhibition of MK3 activity results in altered negative regulatory feedback to MEK/ERK we here show that gain or loss of MK3 function also affects P38. P38 levels appear to be increased by all MK3 variants, including a kinase-defective MK3, suggesting that this effect is independent of its catalytic activity. Interestingly, a number of potential oncogenic MK3 mutations showed distinct effects on ERK and P38 phosphorylation, and in contrast to non-mutant MK3 and MK3 kinase mutants, these mutants did not reduce proliferation capacity of TIG3 cells. Combined our observations support the idea that the effect of MK3 expression level modulation or MK3 mutant is dependent on cellular and genetic context.

### Known senescence checkpoints in relation to MK3 modulation

TP53 and P21^CIP1/WAF1^ levels are increased both in *MK3* ^*WT*^ *OE* and *shMK3* normal cells, in accordance with their pivotal role early in the senescence response [16,40]. The increased expression of P16^INK4a^ upon sustained *MK3* overexpression is in good agreement with its crucial role in establishing irreversible senescence [17,21,22]. As the cancer cell line models used herein are all known to be defective in their P14ARF/TP53 and/or p16/pRB checkpoints, the opposing responses of normal fibroblasts and cancer cells to MK3-depletion suggest an involvement of *CDKN2A/INK4A*. The observation that U-2OS proliferation is negatively affected by gain of MK3 was unexpected in light of the reported epigenetic inactivation of *P16* ^*INK4A*^ in osteosarcoma cells [38,39]. Importantly, however, we show in the present and a previous study [8] that *P14* ^*ARF*^ is induced in senescent U-2OS/*MK3* ^*WT*^ *OE*, which, like P16^INK4A^, is associated with replication checkpoints in human cells [23,41]. Of note, MK2 was recently suggested to control murine haematopoietic stem-cell renewal through P19^ARF^ [42]. Although the mechanism by which *CDKN2A/INK4A* expression in U-2OS/*MK3* ^*WT*^ *OE* cells is activated remains elusive at this time, our findings suggests that P14^ARF^ may be part of the anti-proliferative response in *MK3* ^*WT*^ *OE* cells. In addition, TP53 is induced by *MK3* ^*WT*^ *OE* in both cancer cell lines. Even though P14^ARF^ is known to stabilize TP53 [43], we cannot formally rule out mutually independent roles for TP53, P14^ARF^ and P21^CIP1/WAF1^ in MK3-dependent proliferative control; such independent roles have been reported [44,45].

### MK3 modulation and M/SAPK signaling imbalance

Our previous and current data shows that modulation of MK3 levels causes an imbalance in mitogenic and stress signaling: we find that SAPK P38 and JNK levels are increased in cells overexpressing MK3, whereas loss of MK3 correlates with reduced P38. We recently showed that MK3 controls SAPKs at multiple levels (*i*.*e*. JNK and P38 expression/stabilization) and that MK3 inhibition increased pERK levels in human fibroblasts, which correlated with aberrant expression of immediate early genes (*IEG;* e.g. *ATF3*, *EGR1*) [14]. The finding that MK3 affects P38 and ERK also agrees well with the fact that we established genetic interaction between *dMK2* and *rolled* (dERK) and dP38 in *Drosophila* [14]. MK3 was shown to be targeted by all three canonical M/SAP-kinases [4], which uniquely positions MK3 at the convergence point of potentially conflicting signalling input. We here provide evidence that MK3-overexpression or ablation changes signaling through MAPK and SAPK, and has profound effects on proliferative control both in normal and cancer cell lines, positioning MK3 function both up- and down-stream of M/SAPKs, likely as part of regulatory feedback loops. The involvement of MAPKs and SAPKs in cell proliferation and proliferative life span is well documented [46,47]. Immediate-early response genes represent a standing response mechanism to a variety of triggers; their activation is closely linked to M/SAPK and MK action [48–51]. Our previous studies suggest that altered M/SAPK signalling to IEGs in the context of altered MK3 function plays a vital role in mitogenic and thereby potentially in oncogenic responses [14]. Analogously, the outcome of oncogenic signalling imbalance was reported to be dependent on critical threshold expression levels of the IEG N-MYC and, equally relevant, on the cells’ intrinsic (*i*.*e*. genetic) state and its microenvironment [52]. Similarly, tumor suppressor function has been proposed to involve dosage and context, rather than *all-or-none* type effects [53]. As holds true for M/SAPKs, relative concentrations of MK2, MK3, and MK5 and cellular context have been proposed to dictate protein-protein interactions and thus signalling events and outcome [2,3,49].

### MK3 in tumorigenesis

We used the Cancer Cell Line Encyclopedia (CCLE) to assess MK3 expression in numerous cancer cell lines [32]. This survey revealed that MK3 is relatively highly expressed in various cancer cell types whereas its expression is low/absent in other cancer cell types. *MK3* was originally proposed as a potential tumor-suppressor gene (TSG), as it located in chromosomal region 3p21.3, which frequently carries deletions in cancer. Of interest, *MK3* was recently suggested to harbour potentially oncogenic (driver) mutations, in contrast to *MK2* [7]; these included a series of missense mutations: P28S, E105A and D276Y. The P28S mutation occurs N-terminally to a predicted P-loop (AA 52–57) and the ATP-binding pocket (AA 71–77) at a position which may be conserved between MK2 and MK3 as part of a relatively proline-rich area. The E105A mutation occurs in a group of relatively polar adjacent amino acids, in between the ATP-binding pocket and two regulatory threonines (T201/T313), which constitutively activate MK3 when mutated (TT>EE in MK3^CA^). The D276Y occurs in between the aforementioned regulatory threonines. In the current study we used our normal diploid human fibroblast model to read out effects of such two of these mutations on M/SAPK signalling and cell proliferation. Although the exact role of these mutations in tumorigenesis awaits further analysis, our observations suggest that the MK3^P28S^ and MK3^E105A^ affect cell proliferation and M/SAPK cell signalling via distinct mechanisms compared to MK3^WT^. Expression of both MK3 mutants correlates with a relatively higher pERK level compared to MK3^WT^ or MK3 kinase mutants (KM or CA). Although both novel mutants enhance P38 signalling (level and phosphorylation), and prolonged expression of the MK3^E105A^ form correlates with relatively high P16^INK4A^ expression, neither of the two missense mutants exerts a proliferative disadvantage relative to empty vector control transduced TIG3 cells. The MK3^E105A^ mutant was originally identified in an endometrial carcinoma (EC); of note endometrial cancers show a relatively high mean MK3 expression level among cancer types [32]. The MK3^P28S^ mutant was found in a glioblastoma, a rather heterogeneous cancer type in respect to MK3 expression. Although any statement on the effect of these mutations on MK3 activity would be speculative at this point, it is conceivable that such MK3 mutants result in altered subcellular MK3 interactions that tilt the balance toward mitogenic and/or survival signalling. With respect to the MK3^P28S^ variant an obvious *caveat* is its low expression level in the current study. In depth examination of their biochemical properties, their interactome and their general effect on cell biology in the absence of wild type MK3 would prove useful to fully understand the possible implications of these potential oncogenic MK3 mutations.

The highly homologous MK2 and MK3 proteins have acquired divergent cellular-context dependent functions [2,54]. MK5 (PRAK), although required for RASV12-mediated OIS, is by itself not capable of inducing senescence and is not required for damage-induced responses [55]. We find that MK3 overexpression induces a phenotype reminiscent of oncogene-induced senescence (OIS), as evidenced by accumulation of DNA damage, activation of a DNA damage response, an intraS-phase arrest accompanied by enhanced TP53/P21^CIP1/WAF1^ expression, and ultimately P16^INK4^ and SA-bGal expression and morphological alterations typical of senescent cells. Conversely, MK3 depletion provides a selective growth advantage for cancer cell lines, which fits with enhanced mitogenic signalling through MEK/ERK, and is consistent with loss of TSG function [14]. Although the consequences of MK3 modulation on proliferative life span are likely secondary to abnormal signalling and altered checkpoint activity, combined our findings suggest that both MK3 gain- or loss-of-function may contribute to tumorigenesis depending on cellular and/or genetic context.

### Functional interactions between MK3 and PRC1 in checkpoint control

The MK3-induced proliferative arrest in normal fibroblasts can be overcome by BMI1 co-expression, and clearly correlates with down-regulation of *CDKN2A/P16INK4A* expression, suggesting that MK3 and PRC1 cooperatively control proliferative lifespan. The finding that BMI1 only bypasses the effects of MK3 overexpression, not of MK3 depletion in normal cells, suggests that PRC1-dysfunction (*i*.*e*. complex disruption, inactivation) is unlikely to provide a common PRC1-dependent mechanistic explanation for the proliferative arrest under MK3 gain or loss-of-function. Instead these findings support the involvement of additional, PRC1-independent mechanisms in MK3-mediated checkpoint and replicative lifespan control. We did not observe a bypass-effect of EZH2 overexpression in TIG3/*MK3* ^*WT*^ *OE* cells in this study. EZH2 is implicated in *CDNK2A/INK4A* repression, in stem-cell regulation and senescence [26,31,56–60]. Although published studies and our observations show reduced local H3K27me3 marking at the CDNK2A locus, globally enhanced H3K27me3-chromatin association appears to correlate to physiological stress (*e*.*g*. replication, hypoxia, oxidation stress; unpublished observations JWV). Given the concurrent drop in EZH2 levels and the fact that it fails to bypass MK3-induced senescence, this, by inference, implicates other regulators, among which likely H3K27me3 demethylases, in senescence responses [61]. Regulation of gene expression by histone methyl transferases like EZH2 and demethylases is known to involve regulatory non-coding RNAs, functional association with DNMTs and reciprocal functional interactions with TP53 and/or P16^INK4A^ [28,62–65]. The exact role of EZH2 in bypassing senescence is as yet not clear and may depend on tumor cell type [64,66]. As transcriptional regulatory interdependency among PRC genes has been reported [9], whether or not the reduced PRC1 levels observed in this study represent a hallmark of senescence remains to be established. The combined data herein further supports a functional interaction between PRC1 and MK3. The PRC1 oncoprotein BMI1 may thus control human cell proliferation and differentiation in ways not solely dependent on INK4A, but for instance by repression of proto-oncogenes [67]. In keeping with this idea, BMI1/RAS oncogenic collaboration and RAF1-induced senescence also involve p16^INK4A^–independent mechanisms [68,69].

## Conclusion

In summary, our findings suggest that MK3 controls cell proliferation via multiple pathways, including M/SPAKs, TSGs including TP53, P21^cip1/waf1^, *CDKN2A/INK4A* and PRC1. The effect of gain or loss of MK3 function is context dependent and is likely to be influenced by the large variation of genetic events (heterozygous and homozygous deletions, loss of heterozygocity) involving chromosome 3p and other chromosomes in cancer. Our findings provide a starting point for systematic evaluation of these effects in different cancer types. The finding that MK3-overexpression apparently reactivates dormant checkpoints in cancer cells is an important observation, as it holds the promise of identification of novel therapeutic targets and stresses the relevance of personalized anti-cancer approaches.

## Methods

### Cell lines and cell culture

Human U-2OS osteosarcoma cells [70] expressing the murine ecotropic receptor were kindly provided by D. Shvarts (Utrecht Medical Center, Utrecht, The Netherlands), HeLa cervical carcinoma cells [71] by M. Koritzinsky (MAASTRO, Maastricht, The Netherlands). Normal human fibroblasts BJ [72] and TIG3 [73] were obtained through collaboration; normal human TIG3 fibroblasts expressing the murine ecotropic receptor: courtesy D. Peeper (Netherlands Cancer Institute, Amsterdam, The Netherlands). Cells were cultured under standard conditions in medium supplemented with 10% fetal calf serum and antibiotics (100 units/ml penicillin and 100 μg/ml streptomycin; Gibco). Serum starvation: 0.05% FCS (cancer cell lines) and 0.1% (human fibroblasts) for 48 hrs. Mitogenic stimulation: supplementation of 15% FCS and 100ng/ml tetradecanoyl phorbol acetate (TPA; Sigma-Aldrich, St. Louis, MO, USA) for the indicated duration.

### cDNAs and expression systems

*MK3* ^*KM*^ and *MK3* ^*CA*^ mutant cDNAs were produced by S. Ludwig (Münster, GE). The *MK3* ^*P28S*^ and *MK3* ^*E105A*^ mutants were generated using the QuickChange site-directed mutagenesis method (Agilent Technologies Netherlands B.V.) and sequence verified. Retroviral expression vectors were used to maximize the percentage of expressing cells and to minimize integration effects [74,75]. Retroviral vectors (pBABE-PURO, pBMN-LZRS.ires.GFP, pBMN-LZRS.ires.NEO) expressing murine *Bmi1* and human *MK3* have been described [8]. Expression vectors encoding the murine ecotropic receptor or hTERT and human HA-tagged EZH2 were kindly provided by R. Bernards (Amsterdam, The Netherlands) and T. Jenuwein (Freiburg, Germany), respectively. Criteria used for *shRNA*-sequence design and the retroviral expression system were as described before [76]. Targeting sequences are listed in S1 Table. Transduced cells were selected for 1 week on 4–16 μg/ml puromycin (Sigma).

### Proliferation Assays

Growth curves were standardized: to compare cell genotypes, all viral transductions (*i*.*e*. various expression vectors within one experiment) were performed at the same time; all cells were seeded at equal density (± 20.000 cells/cm2) in 12-multiwell plates (Greiner Bio-one) the preceding evening and allowed to adhere overnight, before the first time point was fixed (t = 0) and followed over time. Cells were collected at the indicated time point cells, washed twice with phosphate-buffered saline and fixed for 10 minutes with 3.7% formaldehyde at room temperature. Next, cells were rinsed 5 times with demineralized water. Cells were stained with 0.1% Chrystal violet for 30 minutes or overnight, and washed 5 times with demineralized water. Chrystal violet was extracted with 10% acetic acid and absorbance was measured spectrophotometrically at 590 nm (Benchmark, Biorad). Data points are based on triplicate measurements within one experiment. Data (cell counts at t = 2 to t = 8) were normalized to cell counts at t = 0 for each transduced cell culture individually. Statistical significance (p<0.05) was determined by two-tailed Student’s t-test and presented relative to the empty vector control. All experiments, including transductions and selections, were repeated at least three times using fresh or frozen viral stocks.

### BrdU-pulse labeling & DNA profiling

TIG3 cells were pulse-labelled with 10 μM BrdU (Sigma) for 45 min. DNA of labelled methanol-fixed cells (-20°C, overnight) was denatured (0.4 mg/ml pepsin (Sigma)/ 0.1 N HCl; 30 min) and incubated (2 N HCl, 30 min, 37°C); pH was readjusted to 7–8 with 0.1 M sodium borate buffer pH 8.5. Cells were incubated with 5 μl anti-BrdU Mab (IIB5, courtesy B. Schutte, Maastricht, The Netherlands; 1–1.5 h, RT); washed and incubated with 5 μl FITC-conjugated anti-mouse Pab (rabbit-anti-mouse FITC, Dako, Glostrup, Denmark; 4°C, overnight) washed, resuspended and stained (100 μg/ml RNAse, 20 μg/ml PI/ PBS, 2–3 hrs, RT, dark) immediately prior to FACS-analysis (Becton Dickinson; CellQuest software).

### Immuno-histochemistry, fluorescent *in situ* hybridization (FISH), cell staining

Cells were seeded at 5–10% confluence on glass slides, infected at low MOI (± 1:1) and subjected to a selection (*i*.*e*. 8 (TIG3) -16 **μ**g (U-2OS) puromycin/ml). Cells were fixed (2% formaldehyde/PBS, 10 min RT), permeabilized (0.2% Triton-X100/PBS) or directly fixed and permeabilized (100% methanol, 20 min, -20°C). Antisera were diluted in blocking buffer (5% normal goat serum, 5% FCS, 0.02% TritonX100/PBS). Polyclonal (Pab) rabbit antiserum glutathione S-transferase (GST) and monoclonal antiserum (Mab) against MK3 were kindly provided by S. Ludwig (Münster, Germany), anti-2Py-tag Mab (MMS-115R; Babco, Richmond, California); anti-p14ARF/p16ß Pab (Ab-1; LabVision corp., Fremont, California), anti-p16INKA mab (E6H4, MTM CINtec laboratories, Heidelberg, Germany), anti-p21 Pab (C-19; Santa Cruz Biotechnology, Santa Cruz, CA, USA), anti-P53 Mab (DO-7; M7001; DAKO), anti-γH2AX Mab (JBW301, 05–636, Upstate Biotechnology/Millipore), anti-pKAP1S824 Pab (A300-767A; Bethyl Laboratories, Montgomery, TX, USA). Primary Mabs were detected with goat anti-mouse TexasRed (TXRD, Southern Biotech, Birmingham, LA, USA), primary Pabs with goat-anti-rabbit fluorescein isothiocyanate (FITC, Southern Biotech). 4’-6-Diamidino-2-phenylindole (DAPI) was co-incubated with secondary conjugated antibodies to counterstain cell nuclei. For karyotypic analysis of TIG3, U-2OS and HeLa cells (S9 Fig), interphase FISH analysis was essentially performed as described [77,78]; Urovysion probe mixture (Abbott Molecular) containing probes for the chromosome 3, 7 and 17 centromeric regions and the 9p21 locus, labelled with SpectrumRed, SpectrumGreen, SpectrumAqua and SpectrumGold, respectively, was used; cells were counter-stained with DAPI. SA-bGal assays were performed as previously described [79].

### Chromatin immunoprecipitation (ChIP) assays

TIG3 cells were transduced with pBABE-puro-GST or GST-3pK and selected with 4μg/ml puromycin. Fixation and ChIP procedures were essentially carried out as described before [14,57]. Briefly, cross-linked fragmented chromatin samples were immuno-precipitated overnight at 4°C with antibodies against either HA (sc-805; Santa Cruz), H3K27me3 (07–449; Upstate), an equal mix of anti-CBX8 "LAST" and "GALD" [57], anti-MK3 [4] or anti-PHC1 [80]. Crosslinks were reversed overnight at 65°C followed by a 2 h digestion with RNAse A and protease K. DNA fragments were recovered using QIAquick PCR purification columns (Qiagen, Hilden, Germany), according to manufacturers’ instructions. Samples were eluted in 75 μl elution buffer and then further 1/5 diluted in TE buffer. Immuno-precipitated DNA was quantified by real-time qPCR. Primer sequences: S1 Table.

### Western analysis

Cell lysis: RIPA buffer (5 mM Benzamidine, 5 μg/ml Antipain, 5 μg/ml Leupeptin, 5 μg/ml Aprotinin, 1 mM Sodium Vanadate, 10 mM Sodium Fluoride, 10 mM Pyrophosphate, 10 mM ß-Glycerophosphate, 0.5 mM DTT and 1 mM PMSF). Protein concentration: BCA protein assay kit (Pierce/Thermo Fisher Scientific, Rockford, IL, USA). Proteins were immobilized on polyvinylidene fluoride (PVDF) membranes, blocked (5% non-fat dry milk/PBS/0.1% Tween-20), incubated with antibodies (4°C, overnight): beta-Actin Mab (C4, 691001, MP Biomedicals, Solon, OH, USA), CBX8 (courtesy K.Hansen, Copenhagen, Denmark), Ezh2 Mab (BD4) and p16 Mab (DCS50; courtesy A. Bracken, BRIC, Copenhagen, Denmark), histone H3 Pab (Ab1791, Abcam, Cambridge, UK), p21 Pab (C-19; Santa Cruz Biotechnology), TP53 Mab (DO-7; M7001; DAKO), anti Bmi1 Mab (F6; courtesy M. vanLohiuzen; Amsterdam, The Netherlands), anti MK3 Mab (3pK; clone 3p8-1, S.Ludwig, Münster, Germany), MK3 Pab (2362 3pK; U.Rapp, Würzburg, Germany), H3K27me3 Pab (07–449, Upstate), 2Py-tag Mab (MMS-115R; Babco), AKT (9272, Cell Signaling, Danvers, MA, USA), EGR1 (sc-110, Santa Cruz), MK2 Pab (3042, Cell Signaling). Signals were detected on autoradiograms using enhanced chemoluminescence (ECL; Pierce).

### Quantification of mRNA levels by real-time (rt) PCR

Messenger NA levels were essentially measured as previously described [14]. Briefly, total RNA was isolated using Tri-Reagent (Sigma). RNA Quantity and quality were measured on the nanodrop (Witec AG, Luzern, Switserland. RNA (500 ng) was converted into cDNA using the iScript cDNA synthesis kit (Bio-Rad, Herculus, CA, USA). MyIQ analysis was performed on 10 ng cDNA using the qPCR iQ Custom SYBR Green Supermix (Bio-Rad) and 300 nM primer. Analysis was performed on an iCycler thermal cycler (Bio-Rad). *Cyclophillin A* was used for normalization in all experiments. Primers sequences: S1 Table.

## Supporting Information

S1 Fig *MK3* overexpression induces a proliferative arrest in normal human fibroblasts. (A) Representative BrdU/DNA profile of TIG3 cells expression *MK3* ^*WT*^ (*MK3* ^*WT*^ *OE*; lower panel) or control cells (con; upper panel). Cells were analyzed for cell cycle distribution approximately 1 week after retroviral transduction and selection. (B) Quantification of relative S-phase distribution in TIG3/*con* and TIG3/*MK3* ^*WT*^ *OE* cells. S-phase sub-stage was assessed based on BrdU-incorporation pattern (*i*.*e*. global or focal) and DAPI staining-intensity (i.e. dim: early S, bright: late S; lower panel): Ed: early S/BrdU-dim, Eb: early S/BrdU-bright, M: mid S, L late S (Ld: late S/BrdU-dim in bottom panel); data are expressed as percentage of the total number of BrdU-positive cells. Note: the nearly even distribution of BrdU-positive cells over the entire S-phase and the relatively low BrdU incorporation of a substantial number of TIG3/*MK3* ^*WT*^ *OE* nuclei are both indicative of intra-S phase arrest. (C) Cell morphology of TIG3^*hTERT*^/*con* cells (top panel) and TIG3^*hTERT*^/*MK3* ^*WT*^ *OE* cells (bottom panel); phase contrast images.
(TIF)

S2 Fig Proliferation capacity of MK3-depleted and MK3-kinase mutant TIG3 cells. (A) Proliferation profiles of TIG3/*MK3* ^*KM*^ *OE* and TIG3/*MK3* ^*CA*^ *OE* cells at 1 week (left panel) and ±4 weeks post-transduction (right panel). Note: proliferation profiles for all cell genotypes depicted in graphs S2A Fig and Fig 6B were derived simultaneously: cells were synchronously retrovirally transduced, selected, expanded and plated to determine proliferation characteristics. Cell counts at t = 2 through t = 8 were normalized to cell counts at t = 0 for each transduced cell culture individually (see Methods section for details); statistical significance was determined by two-tailed Student’s t-test and is presented relative to the empty vector control (* *p* < 0.05). (B) Protein expression profiles of GST-tagged MK3 (GST:MK3), TP53 and p16^INK4A^ (P16) in TIG3 cells at indicated time points post-transduction (corresponding to Fig 2SA); loading controls: b-Actin (bAct). Note: the Immunoblot analysis depicted is part of the analysis as presented in Fig 6C. (C) Immunoblot analysis of two different short hairpin-based RNAi vectors targeting MK3 only (*shMK3*) or MK2 and MK3 simultaneously (*shMK2/3*); loading control Tubulin (Tub). (D) Representative DNA profiles (BrdU pulse-labeling and S-phase quantification by FACS) of TIG3/*shMK3* cells corresponding to Fig 4B. (E) Quantification of DNA profiles (BrdU pulse-labeling and S-phase quantification by FACS) in TIG3/*shMK3 and* TIG3/*con* cells at approximately 1 week post-transduction.
(TIF)

S3 Fig Functional interactions between MK3 and Polycomb Group proteins. (A) Morphology of cell cultures corresponding to the chromatin immunoprecipitation (ChIP) experiments (Fig 3C); phase contrast images (right panels) confirm flat cell phenotype in TIG3/*MK3* ^*WT*^ *OE* cells at the time of harvest. (B) Real-time PCR analysis of *EZH2* and *INK4A/ARF* mRNA levels in TIG3/*MK3* ^*WT*^ *OE* and control cells; bottom: schematic overview of human *INK4A/ARF* locus and primers used in this study. Due to overlap in mRNA sequences of *p14* ^*ARF*^ and *p16* ^*INK4A*^, levels of *p16* ^*INK4A*^ mRNA could not be directly measured by real time PCR; instead mRNA levels for *p14* ^*ARF*^ only, and for *p14* ^*ARF*^ + *p16* ^*INK4A*^ were measured; *p16* ^*INK4A*^ mRNA levels were deduced by subtraction; error bars are provided for *p14* ^*ARF*^ and for *p14* ^*ARF*^ + *p16* ^*INK4A*^ measurements.
(TIF)

S4 Fig Functional interaction between MK3 and Polycomb Repressive Complex 1. (A) Analysis of P16^INK4A^ (P16) levels in control (con), HA-tagged EZH2 (HA:EZH2); 2PY-tagged BMI1 (BMI1:2PY) and GST-tagged MK3 (GST:MK3)-transduced TIG3 cells in the presence or absence of *MK3* ^*WT*^ *OE (*GST:MK3*);* tubulin (tub) and histone H3 (H3) represent fractionation controls. Cytoplasmic and nuclear fractions were always loaded on the same gel for protein analysis (corresponding sections are shown separately); nuclear fractions correspond to approximately 3–4 cytoplasmic equivalents; antibodies used as indicated in figure. (B) Real-time quantitation of mRNA expression in TIG3 cells; mRNAs as indicated; note that the exon2 primer-set simultaneously detects P14^ARF^ and P16^INK4A^ (*cf*. S3 Fig) Expression data were normalized to cyclophillin expression; shRNA vectors used are indicated below the graph.
(TIF)

S5 Fig MK3 expression levels in normal and cancer cell lines. (A) Expression levels of MK3 mRNA based on analysis of nearly 1000 cancer cell lines (source: *Broad-Novartis Cancer Cell Line Encyclopedia*; http://www.broadinstitute.org/ccle [32]). (B) MK3 and MK2 protein expression in normal and cancer cell lines; TIG3: diploid human fibroblast; BJ2: diploid human fibroblast; HeLa: cervical carcinoma; U-2OS: osteosarcoma; HCT116, HT29: colorectal carcinoma; DU145: prostate carcinoma; MCF-7: breast carcinoma; U251, U87, U373 glioblastoma; A549: alveolar adeno-carcinoma; ECC1, Ishikawa: endometrial carcinoma; RL95-2 cervical adenosquamous carcinoma.
(TIF)

S6 Fig Flat cell formation in U-2OS/*MK3* ^*WT*^ *OE* cultures. (A) Confirmation of MK3^*WT*^ expression in flat U-2OS/*MK3* ^*WT*^ *iresGFP* cells (upper left panel; phase contrast) transduced with a retroviral vector co-expressing GST:MK3 and GFP (lower left panel); right panels: large flat GFP-positive U-2OS cells stain positive for SA-bGal; arrows demarcate flat cells positive for GFP (upper panel) and SA-bGal (lower panel). (B) TP53 and P14^ARF^ (P14) co-staining (left panels) or P14^ARF^ (P14) and P21^CIP1/WAF1^ (P21) co-staining (right panels) in senescent U-2OS/*MK3* ^*WT*^ *OE* cells; nuclei were counterstained with DAPI.
(TIF)

S7 Fig Modulation of MK3-levels causes signalling imbalance. (A) Immunoblot analysis of M/SAPK (ERK, P38, JNK) and phosphorylated (pERK, pP38) levels in TIG3/*MK3* ^*WT*^ *OE* (*MK3* ^*WT*^ *OE*) cells *versus* control cells (con). (B) Immunoblot detection of sustained elevated P38 levels in TIG3/*MK3* ^*WT*^ *OE* cells between 1 and 3 weeks post transduction. (C) Immunoblot detection of P38 levels in TIG3/*MK3* ^*WT*^ *OE*, U-2OS/*MK3* ^*WT*^ *OE* and HeLa/*MK3* ^*WT*^ *OE* cells. (D) Immunoblot detection of P38, l TP53, P21^cip1/waf1^ (P21) and p16^INK4A^ (P16) protein expression levels; loading controls: b-Actin (bAct). (E) Left: morphological changes in U-2OS/*MK3* ^*WT*^ *OE* cultures under reduced serum conditions (1%); empty vector control cells (*con*). Right: quantitation of serum-deprivation on proliferative capacity of U-2OS/*MK3* ^*WT*^ *OE* cells (squares: 10% FCS; circles: 5% FCS; triangles: 2% FCS; open symbols: control; filled symbols: *MK3OE*). Indicated (brackets) is percentage growth reduction relative to control cells at each serum concentrations.
(TIF)

S8 Fig Modulation of MK3-levels causes signalling imbalance. (A) Immunoblot analysis of P38 and phosphorylated P38 (pP38) levels in serum-starved/stimulated TIG3/*MK3* ^*WT*^ *OE* (overexpression of wild type MK3; *WT*), TIG3/*MK* ^*K*M^OE (kinase dead MK3; *KM*), TIG3/*MK3* ^*CA*^ *OE (*constitutively active MK3; *CA*), TIG3/*MK3* ^*P28S*^ *OE* (potential oncogenic mutation P28S; P28S) or TIG3/*MK3* ^*E105A*^ *OE* (potential oncogenic mutation E105A; E105A); *con* represents empty retroviral vector control cells. All cells were synchronously transduced, selected, serum starved and serum/TPA stimulated. Extracts were prepared at the indicated time points (time in minutes). Loading control: Tubulin (Tub); “t” (GST:MK3 panel) refers to longer exposure time of autoradiographic film. (B) Immunoblot analysis of P38 and pP38 levels at defined time points in the same extracts as (A); extracts of all TIG3/*MK3*-genotypes at defined time points were loaded on the same blot to facilitate direct quantitative comparison.
(TIF)

S9 Fig Karyotypic analysis of used cell lines. (A) Representative FISH image of TIG3 (left), U-2OS (middle) and HeLa cells (right); detection of 9p21 (*CDKN2A/INK4A locus*, yellow) and chromosome 17 centromeres (blue); DAPI was used to counterstain nuclei. (B) Upper table depicts summary of limited karyotype analysis; lower table depicts full analysis of TIG3 (n = 67; >98% diploid), U-2OS (n = 102; mixed polyploidy) and HeLa (n = 100; >90% triploidy at all loci investigated) nuclei for centromeres of chromosome 3, 7 and 17 and 9p21.
(TIF)

S1 Table Primers used for ChIP and mRNA expression analysis. (DOCX)

## References

[1] Keshet Y, Seger R. The MAP kinase signaling cascades: a system of hundreds of components regulates a diverse array of physiological functions. Methods Mol Biol. 2010; 661: 3–38. 10.1007/978-1-60761-795-2_1 20811974

[2] Gaestel M. MAPKAP kinases—MKs—two's company, three's a crowd. Nat Rev Mol Cell Biol. 2006; 7: 120–130. 16421520 10.1038/nrm1834

[3] Shiryaev A, Moens U. Mitogen-activated protein kinase p38 and MK2, MK3 and MK5: menage a trois or menage a quatre? Cell Signal. 2010; 22: 1185–1192. 10.1016/j.cellsig.2010.03.002 20227494

[4] Ludwig S, Engel K, Hoffmeyer A, Sithanandam G, Neufeld B, Palm D, et al 3pK, a novel mitogen-activated protein (MAP) kinase-activated protein kinase, is targeted by three MAP kinase pathways. Mol Cell Biol. 1996; 16: 6687–6697. 8943323 10.1128/mcb.16.12.6687 PMC231671

[5] Protopopov AI, Li J, Winberg G, Gizatullin RZ, Kashuba VI, Klein G, et al Human cell lines engineered for tetracycline-regulated expression of tumor suppressor candidate genes from a frequently affected chromosomal region, 3p21. J Gene Med. 2002; 4: 397–406. 12124982 10.1002/jgm.283

[6] Sithanandam G, Latif F, Duh F, Bernal R, Smola U, Li H, et al 3pK, a new mitogen-activated protein kinase-activated protein kinase located in the small cell lung cancer tumor suppressor gene region [published erratum appears in Mol Cell Biol 1996 Apr;16(4):1880]. Mol Cell Biol. 1996; 16: 868–876. 8622688 10.1128/mcb.16.3.868 PMC231067

[7] Greenman C, Stephens P, Smith R, Dalgliesh GL, Hunter C, Bignell G, et al Patterns of somatic mutation in human cancer genomes. Nature. 2007; 446: 153–158. 17344846 10.1038/nature05610 PMC2712719

[8] Voncken JW, Niessen H, Neufeld B, Rennefahrt U, Dahlmans V, Kubben N, et al MAPKAP kinase 3pK phosphorylates and regulates chromatin association of the polycomb group protein Bmi1. J Biol Chem. 2005; 280: 5178–5187. 15563468 10.1074/jbc.M407155200

[9] Prezioso C, Orlando V. Polycomb proteins in mammalian cell differentiation and plasticity. FEBS Letters. 2011; 585: 2067–2077. 10.1016/j.febslet.2011.04.062 21575638

[10] Niessen HE, Demmers JA, Voncken JW. Talking to chromatin: post-translational modulation of polycomb group function. Epigenetics Chromatin. 2009; 2: 10 10.1186/1756-8935-2-10 19723311 PMC2745409

[11] Simon JA, Kingston RE. Mechanisms of Polycomb gene silencing: knowns and unknowns. Nat Rev Mol Cell Biol. 2009; 10: 697–708. 10.1038/nrm2763 19738629

[12] Voncken JW, Schweizer D, Aagaard L, Sattler L, Jantsch MF, van Lohuizen M. Chromatin-association of the Polycomb group protein BMI1 is cell cycle- regulated and correlates with its phosphorylation status. J Cell Sci. 1999; 112: 4627–4639. 10574711 10.1242/jcs.112.24.4627

[13] Sparmann A, van Lohuizen M. Polycomb silencers control cell fate, development and cancer. Nat Rev Cancer. 2006; 6: 846–856. 17060944 10.1038/nrc1991

[14] Prickaerts P, Niessen HEC, Mouchel-Vielh E, Dahlmans VEH, van den Akker GGH, Geijselaers C, et al MK3 controls Polycomb target gene expression via negative feedback on ERK. Epigenetics Chromatin. 2012; 5: 12 10.1186/1756-8935-5-12 22870894 PMC3499388

[15] Ronkina N, Kotlyarov A, Dittrich-Breiholz O, Kracht M, Hitti E, Milarski K, et al The mitogen-activated protein kinase (MAPK)-activated protein kinases MK2 and MK3 cooperate in stimulation of tumor necrosis factor biosynthesis and stabilization of p38 MAPK. Mol Cell Biol. 2007; 27: 170–181. 17030606 10.1128/MCB.01456-06 PMC1800641

[16] Serrano M, Lin AW, McCurrach ME, Beach D, Lowe SW. Oncogenic ras provokes premature cell senescence associated with accumulation of p53 and p16INK4a. Cell. 1997; 88: 593–602. 9054499 10.1016/s0092-8674(00)81902-9

[17] Campisi J. Senescent cells, tumor suppression, and organismal aging: good citizens, bad neighbors. Cell. 2005; 120: 513–522. 15734683 10.1016/j.cell.2005.02.003

[18] Courtois-Cox S, Genther Williams SM, Reczek EE, Johnson BW, McGillicuddy LT, Johannessen CM, et al A negative feedback signaling network underlies oncogene-induced senescence. Cancer Cell. 2006; 10: 459–472. 17157787 10.1016/j.ccr.2006.10.003 PMC2692661

[19] Halazonetis TD, Gorgoulis VG, Bartek J. An oncogene-induced DNA damage model for cancer development. Science. 2008; 319: 1352–1355. 10.1126/science.1140735 18323444

[20] Yaswen P, Campisi J. Oncogene-Induced Senescence Pathways Weave an Intricate Tapestry. Cell. 2007; 128: 233–234. 17254959 10.1016/j.cell.2007.01.005

[21] Beausejour CM, Krtolica A, Galimi F, Narita M, Lowe SW, Yaswen P, et al Reversal of human cellular senescence: roles of the p53 and p16 pathways. Embo J. 2003; 22: 4212–4222. 12912919 10.1093/emboj/cdg417 PMC175806

[22] Collado M, Blasco MA, Serrano M. Cellular senescence in cancer and aging. Cell. 2007; 130: 223–233. 17662938 10.1016/j.cell.2007.07.003

[23] Campisi J. Cellular senescence: putting the paradoxes in perspective. Current Opinion in Genetics & Development. 2010; 21: 107–112. 21093253 10.1016/j.gde.2010.10.005 PMC3073609

[24] Prieur A, Peeper DS. Cellular senescence in vivo: a barrier to tumorigenesis. Curr Opin Cell Biol. 2008; 20: 150–155. 10.1016/j.ceb.2008.01.007 18353625

[25] Bracken AP, Kleine-Kohlbrecher D, Dietrich N, Pasini D, Gargiulo G, Beekman C, et al The Polycomb group proteins bind throughout the INK4A-ARF locus and are disassociated in senescent cells. Genes Dev. 2007; 21: 525–530. 17344414 10.1101/gad.415507 PMC1820894

[26] Kheradmand Kia S, Solaimani Kartalaei P, Farahbakhshian E, Pourfarzad F, von Lindern M, Verrijzer CP. EZH2-dependent chromatin looping controls INK4a and INK4b, but not ARF, during human progenitor cell differentiation and cellular senescence. Epigenetics & Chromatin. 2009; 2: 16. 19954516 10.1186/1756-8935-2-16 PMC3225837

[27] Cao R, Zhang Y. The functions of E(Z)/EZH2-mediated methylation of lysine 27 in histone H3. Current Opinion in Genetics & Development. 2004; 14: 155–164. 15196462 10.1016/j.gde.2004.02.001

[28] Tang X, Milyavsky M, Shats I, Erez N, Goldfinger N, Rotter V. Activated p53 suppresses the histone methyltransferase EZH2 gene. Oncogene. 2004. 10.1038/sj.onc.1207706 15208672

[29] Dietrich N, Bracken AP, Trinh E, Schjerling CK, Koseki H, Rappsilber J, et al Bypass of senescence by the polycomb group protein CBX8 through direct binding to the INK4A-ARF locus. Embo J. 2007; 26: 1637–1648. 17332741 10.1038/sj.emboj.7601632 PMC1829390

[30] Gil J, Peters G. Regulation of the INK4b-ARF-INK4a tumour suppressor locus: all for one or one for all. Nat Rev Mol Cell Biol. 2006; 7: 667–677. 16921403 10.1038/nrm1987

[31] Isono K, Fujimura Y, Shinga J, Yamaki M, J OW, Takihara Y, et al Mammalian polyhomeotic homologues phc2 and phc1 act in synergy to mediate polycomb repression of hox genes. Mol Cell Biol. 2005; 25: 6694–6706. 16024804 10.1128/MCB.25.15.6694-6706.2005 PMC1190356

[32] Barretina J, Caponigro G, Stransky N, Venkatesan K, Margolin AA, Kim S, et al The Cancer Cell Line Encyclopedia enables predictive modelling of anticancer drug sensitivity. Nature. 2012; 483: 603–607. 10.1038/nature11003 22460905 PMC3320027

[33] Ehlting C, Ronkina N, Bohmer O, Albrecht U, Bode KA, Lang KS, et al Distinct functions of the mitogen-activated protein kinase-activated protein (MAPKAP) kinases MK2 and MK3: MK2 mediates lipopolysaccharide-induced signal transducers and activators of transcription 3 (STAT3) activation by preventing negative regulatory effects of MK3. J Biol Chem. 2011; 286: 24113–24124. 10.1074/jbc.M111.235275 21586572 PMC3129192

[34] Kotlyarov A, Yannoni Y, Fritz S, Laass K, Telliez JB, Pitman D, et al Distinct cellular functions of MK2. Mol Cell Biol. 2002; 22: 4827–4835. 12052889 10.1128/MCB.22.13.4827-4835.2002 PMC133920

[35] Di Micco R, Fumagalli M, d'Adda di Fagagna F. Breaking news: high-speed race ends in arrest—how oncogenes induce senescence. Trends in Cell Biology. 2007; 17: 529–536. 17980599 10.1016/j.tcb.2007.07.012

[36] Mallette FA, Gaumont-Leclerc M-F, Ferbeyre G. The DNA damage signaling pathway is a critical mediator of oncogene-induced senescence. Genes Dev. 2007; 21: 43–48. 17210786 10.1101/gad.1487307 PMC1759898

[37] Munger K, Scheffner M, Huibregtse JM, Howley PM. Interactions of HPV E6 and E7 oncoproteins with tumour suppressor gene products. Cancer surveys. 1992; 12: 197–217. 1322242

[38] Stott FJ, Bates S, James MC, McConnell BB, Starborg M, Brookes S, et al The alternative product from the human CDKN2A locus, p14(ARF), participates in a regulatory feedback loop with p53 and MDM2. Embo J. 1998; 17: 5001–5014. 9724636 10.1093/emboj/17.17.5001 PMC1170828

[39] Park YB, Park MJ, Kimura K, Shimizu K, Lee SH, Yokota J. Alterations in the INK4a/ARF locus and their effects on the growth of human osteosarcoma cell lines. Cancer Genet Cytogenet. 2002; 133: 105–111. 11943335 10.1016/s0165-4608(01)00575-1

[40] Chen J, Huang X, Halicka D, Brodsky S, Avram A, Eskander J, et al Contribution of p16INK4a and p21CIP1 pathways to induction of premature senescence of human endothelial cells: permissive role of p53. Am J Physiol Heart Circ Physiol. 2006; 290: H1575–1586. 16243918 10.1152/ajpheart.00364.2005

[41] Sherr CJ. The INK4a/ARF network in tumour suppression. Nat Rev Mol Cell Biol. 2001; 2: 731–737. 11584300 10.1038/35096061

[42] Schwermann J, Rathinam C, Schubert M, Schumacher S, Noyan F, Koseki H, et al. MAPKAP kinase MK2 maintains self-renewal capacity of haematopoietic stem cells. Embo J. 2009. 10.1038/emboj.2009.100 PMC2688528 19369945

[43] Pomerantz J, Schreiber-Agus N, Liegeois NJ, Silverman A, Alland L, Chin L, et al The Ink4a tumor suppressor gene product, p19Arf, interacts with MDM2 and neutralizes MDM2's inhibition of p53. Cell. 1998; 92: 713–723. 9529248 10.1016/s0092-8674(00)81400-2

[44] Rowland BD, Peeper DS. KLF4, p21 and context-dependent opposing forces in cancer. Nat Rev Cancer. 2006; 6: 11–23. 16372018 10.1038/nrc1780

[45] Sherr CJ. Divorcing ARF and p53: an unsettled case. Nat Rev Cancer. 2006; 6: 663–673. 16915296 10.1038/nrc1954

[46] Debacq-Chainiaux F, Boilan E, Dedessus Le Moutier J, Weemaels G, Toussaint O. p38(MAPK) in the senescence of human and murine fibroblasts. Adv Exp Med Biol. 2010; 694: 126–137. 20886761 10.1007/978-1-4419-7002-2_10

[47] Maruyama J, Naguro I, Takeda K, Ichijo H. Stress-activated MAP kinase cascades in cellular senescence. Curr Med Chem. 2009; 16: 1229–1235. 19355881 10.2174/092986709787846613

[48] Dunn KL, Espino PS, Drobic B, He S, Davie JR. The Ras-MAPK signal transduction pathway, cancer and chromatin remodeling. Biochem Cell Biol. 2005; 83: 1–14. 15746962 10.1139/o04-121

[49] Murphy LO, Blenis J. MAPK signal specificity: the right place at the right time. Trends in Biochemical Sciences. 2006; 31: 268–275. 16603362 10.1016/j.tibs.2006.03.009

[50] Plotnikov A, Zehorai E, Procaccia S, Seger R. The MAPK cascades: Signaling components, nuclear roles and mechanisms of nuclear translocation. Biochimica et Biophysica Acta (BBA)—Molecular Cell Research. 2011; 1813: 1619–1633. 21167873 10.1016/j.bbamcr.2010.12.012

[51] Sng JC, Taniura H, Yoneda Y. A tale of early response genes. Biol Pharm Bull. 2004; 27: 606–612. 15133230 10.1248/bpb.27.606

[52] Murphy DJ, Junttila MR, Pouyet L, Karnezis A, Shchors K, Bui DA, et al Distinct Thresholds Govern Myc's Biological Output In Vivo. Cancer Cell. 2008; 14: 447–457. 10.1016/j.ccr.2008.10.018 19061836 PMC2723751

[53] Berger AH, Knudson AG, Pandolfi PP. A continuum model for tumour suppression. Nature. 2011; 476: 163–169. 10.1038/nature10275 21833082 PMC3206311

[54] Ronkina N, Kotlyarov A, Gaestel M. MK2 and MK3—a pair of isoenzymes? Front Biosci. 2008; 13: 5511–5521. 18508601 10.2741/3095

[55] Sun P, Yoshizuka N, New L, Moser BA, Li Y, Liao R, et al PRAK is essential for ras-induced senescence and tumor suppression. Cell. 2007; 128: 295–308. 17254968 10.1016/j.cell.2006.11.050

[56] Agherbi H, Gaussmann-Wenger A, Verthuy C, Chasson L, Serrano M, Djabali M. Polycomb mediated epigenetic silencing and replication timing at the INK4a/ARF locus during senescence. PloS one. 2009; 4: e5622 10.1371/journal.pone.0005622 19462008 PMC2680618

[57] Bracken AP, Dietrich N, Pasini D, Hansen KH, Helin K. Genome-wide mapping of Polycomb target genes unravels their roles in cell fate transitions. Genes Dev. 2006; 20: 1123–1136. 16618801 10.1101/gad.381706 PMC1472472

[58] Bracken AP, Pasini D, Capra M, Prosperini E, Colli E, Helin K. EZH2 is downstream of the pRB-E2F pathway, essential for proliferation and amplified in cancer. Embo J. 2003; 22: 5323–5335. 14532106 10.1093/emboj/cdg542 PMC213796

[59] Cakouros D, Isenmann S, Cooper L, Zannettino A, Anderson P, Glackin C, et al Twist-1 induces Ezh2 recruitment regulating histone methylation along the Ink4A/Arf locus in mesenchymal stem cells. Molecular and cellular biology. 2012; 32: 1433–1441. 10.1128/MCB.06315-11 22290439 PMC3318575

[60] Itahana K, Zou Y, Itahana Y, Martinez JL, Beausejour C, Jacobs JJ, et al Control of the replicative life span of human fibroblasts by p16 and the polycomb protein Bmi-1. Mol Cell Biol. 2003; 23: 389–401. 12482990 10.1128/MCB.23.1.389-401.2003 PMC140680

[61] Agger K, Cloos PA, Rudkjaer L, Williams K, Andersen G, Christensen J, et al The H3K27me3 demethylase JMJD3 contributes to the activation of the INK4A-ARF locus in response to oncogene- and stress-induced senescence. Genes Dev. 2009; 23: 1171–1176. 10.1101/gad.510809 19451217 PMC2685535

[62] Reynolds PA, Sigaroudinia M, Zardo G, Wilson MB, Benton GM, Miller CJ, et al. Tumor suppressor P16INK4A regulates polycomb-mediated DNA hypermethylation in human mammary epithelial cells. J Biol Chem. 2006. 10.1074/jbc.M604175200 16766534

[63] So A-Y, Jung J-W, Lee S, Kim H-S, Kang K-S. DNA Methyltransferase Controls Stem Cell Aging by Regulating BMI1 and EZH2 through MicroRNAs. PLoS ONE. 2011; 6: e19503 10.1371/journal.pone.0019503 21572997 PMC3091856

[64] Tzatsos A, Paskaleva P, Lymperi S, Contino G, Stoykova S, Chen Z, et al Lysine-specific demethylase 2B (KDM2B)-let-7-enhancer of zester homolog 2 (EZH2) pathway regulates cell cycle progression and senescence in primary cells. J Biol Chem. 2011; 286: 33061–33069. 10.1074/jbc.M111.257667 21757686 PMC3190920

[65] Vire E, Brenner C, Deplus R, Blanchon L, Fraga M, Didelot C, et al. The Polycomb group protein EZH2 directly controls DNA methylation. Nature. 2005. 10.1038/nature04431 16357870

[66] Fan T, Jiang S, Chung N, Alikhan A, Ni C, Lee CC, et al EZH2-dependent suppression of a cellular senescence phenotype in melanoma cells by inhibition of p21/CDKN1A expression. Molecular cancer research: MCR. 2011; 9: 418–429. 10.1158/1541-7786.MCR-10-0511 21383005 PMC3078218

[67] Oguro H, Yuan J, Tanaka S, Miyagi S, Mochizuki-Kashio M, Ichikawa H, et al Lethal myelofibrosis induced by Bmi1-deficient hematopoietic cells unveils a tumor suppressor function of the polycomb group genes. The Journal of Experimental Medicine. 2012; 209: 445–454. 10.1084/jem.20111709 22351929 PMC3302226

[68] Datta S, Hoenerhoff MJ, Bommi P, Sainger R, Guo W-J, Dimri M, et al Bmi-1 Cooperates with H-Ras to Transform Human Mammary Epithelial Cells via Dysregulation of Multiple Growth-Regulatory Pathways. Cancer Res. 2007; 67: 10286–10295. 17974970 10.1158/0008-5472.CAN-07-1636 PMC2424172

[69] Olsen CL, Gardie B, Yaswen P, Stampfer MR. Raf-1-induced growth arrest in human mammary epithelial cells is p16-independent and is overcome in immortal cells during conversion. Oncogene. 2002; 21: 6328–6339. 12214273 10.1038/sj.onc.1205780

[70] Bruland OS, Fodstad O, Stenwig AE, Pihl A. Expression and characteristics of a novel human osteosarcoma-associated cell surface antigen. Cancer Res. 1988; 48: 5302–5309. 2457439

[71] Scherer WF, Syverton JT, Gey GO. Studies on the propagation in vitro of poliomyelitis viruses. IV. Viral multiplication in a stable strain of human malignant epithelial cells (strain HeLa) derived from an epidermoid carcinoma of the cervix. The Journal of experimental medicine. 1953; 97: 695–710. 13052828 10.1084/jem.97.5.695 PMC2136303

[72] Sitte N, Merker K, Von Zglinicki T, Grune T, DAVIES KJA. Protein oxidation and degradation during cellular senescence of human BJ fibroblasts: part I—effects of proliferative senescence. The FASEB Journal. 2000; 14: 2495–2502. 11099467 10.1096/fj.00-0209com

[73] Baker Darren J, Weaver Robbyn L, van Deursen Jan M. p21 Both Attenuates and Drives Senescence and Aging in BubR1 Progeroid Mice. Cell Reports. 2013; 3: 1164–1174. 10.1016/j.celrep.2013.03.028 23602569 PMC3785294

[74] Kinsella TM, Nolan GP. Episomal vectors rapidly and stably produce high-titer recombinant retrovirus. HumGene Ther. 1996; 7: 1405–1413. 8844199 10.1089/hum.1996.7.12-1405

[75] Morgenstern JP, Land H. Advanced mammalian gene transfer: high titre retroviral vectors with multiple drug selection markers and a complementary helper-free packaging cell line. Nucleic Acids Res. 1990; 18: 3587–3596. 2194165 10.1093/nar/18.12.3587 PMC331014

[76] Brummelkamp TR, Bernards R, Agami R. A system for stable expression of short interfering RNAs in mammalian cells. Science. 2002; 296: 550–553. 11910072 10.1126/science.1068999

[77] Huysentruyt CJ, Baldewijns MM, Ruland AM, Tonk RJ, Vervoort PS, Smits KM, et al Modified UroVysion scoring criteria increase the urothelial carcinoma detection rate in cases of equivocal urinary cytology. Histopathology. 2011; 58: 1048–1053. 10.1111/j.1365-2559.2011.03859.x 21707706

[78] Speel EJ, Schutte B, Wiegant J, Ramaekers FC, Hopman AH. A novel fluorescence detection method for in situ hybridization, based on the alkaline phosphatase-fast red reaction. J Histochem Cytochem. 1992; 40: 1299–1308. 1506667 10.1177/40.9.1506667

[79] Dimri GP, Lee X, Basile G, Acosta M, Scott G, Roskelley C, et al A biomarker that identifies senescent human cells in culture and in aging skin in vivo. Proc Natl Acad Sci U S A. 1995; 92: 9363–9367. 7568133 10.1073/pnas.92.20.9363 PMC40985

[80] Takihara Y, Tomotsune D, Shirai M, Katoh-Fukui Y, Nishii K, Motaleb MA, et al Targeted disruption of the mouse homologue of the Drosophila polyhomeotic gene leads to altered anteroposterior patterning and neural crest defects. Development. 1997; 124: 3673–3682. 9367423 10.1242/dev.124.19.3673

