# Supplementary figures and images for: MK3 Modulation Affects BMI1-Dependent and Independent Cell Cycle Check-Points
Source: PLoS One. 2015 Apr 8;10(4):e0118840. doi: 10.1371/journal.pone.0118840 (PMC4390245; doi:10.1371/journal.pone.0118840)

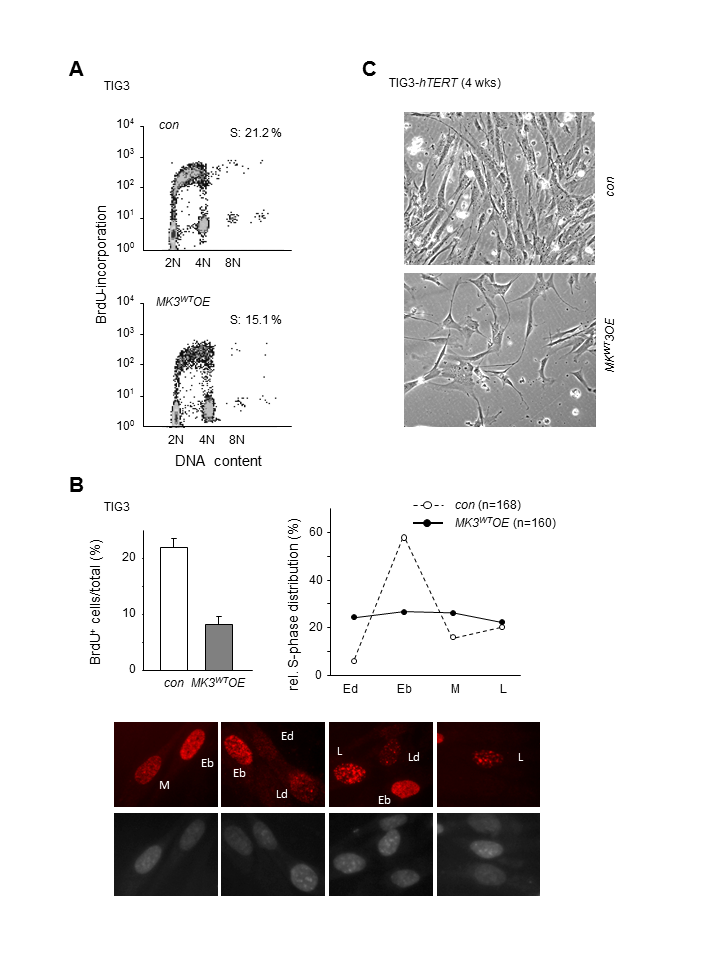

Supplement: S1 Fig — (A) Representative BrdU/DNA profile of TIG3 cells expression MK3 WT (MK3 WT OE; lower panel) or control cells (con; upper panel). Cells were analyzed for cell cycle distribution approximately 1 week after retroviral transduction and selection. (B) Quantification of relative S-phase distribution in TIG3/con and TIG3/MK3 WT OE cells. S-phase sub-stage was assessed based on BrdU-incorporation pattern (i.e. global or focal) and DAPI staining-intensity (i.e. dim: early S, bright: late S; lower panel): Ed: early S/BrdU-dim, Eb: early S/BrdU-bright, M: mid S, L late S (Ld: late S/BrdU-dim in bottom panel); data are expressed as percentage of the total number of BrdU-positive cells. Note: the nearly even distribution of BrdU-positive cells over the entire S-phase and the relatively low BrdU incorporation of a substantial number of TIG3/MK3 WT OE nuclei are both indicative of intra-S phase arrest. (C) Cell morphology of TIG3hTERT/con cells (top panel) and TIG3hTERT/MK3 WT OE cells (bottom panel); phase contrast images. (TIF) [file pone.0118840.s001.TIF]

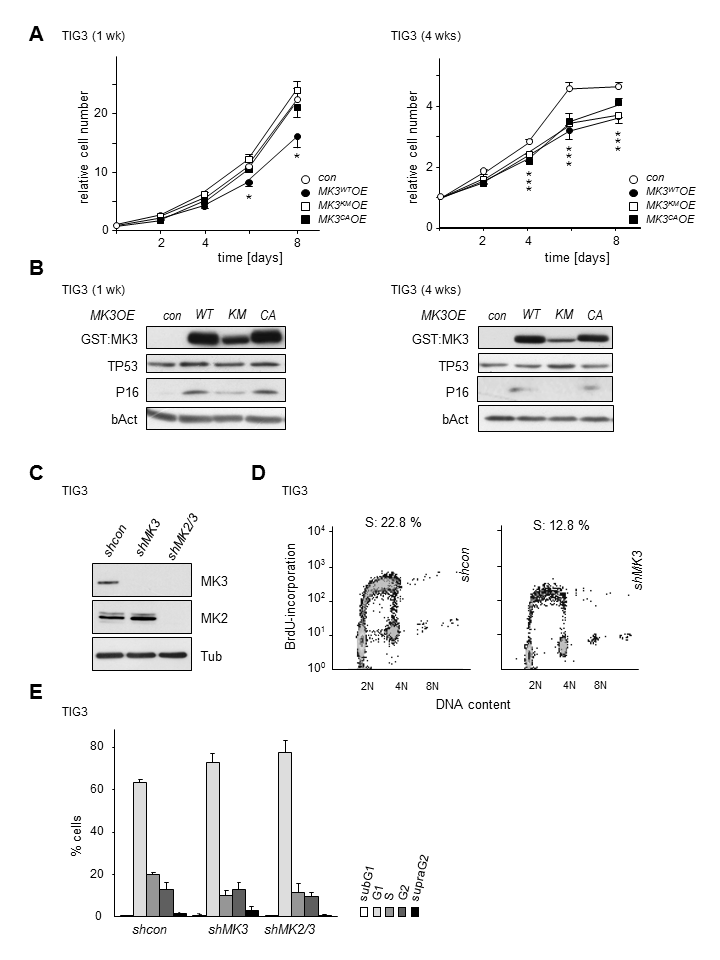

Supplement: S2 Fig — (A) Proliferation profiles of TIG3/MK3 KM OE and TIG3/MK3 CA OE cells at 1 week (left panel) and ±4 weeks post-transduction (right panel). Note: proliferation profiles for all cell genotypes depicted in graphs S2A Fig and Fig 6B were derived simultaneously: cells were synchronously retrovirally transduced, selected, expanded and plated to determine proliferation characteristics. Cell counts at t = 2 through t = 8 were normalized to cell counts at t = 0 for each transduced cell culture individually (see Methods section for details); statistical significance was determined by two-tailed Student’s t-test and is presented relative to the empty vector control (* p < 0.05). (B) Protein expression profiles of GST-tagged MK3 (GST:MK3), TP53 and p16INK4A (P16) in TIG3 cells at indicated time points post-transduction (corresponding to Fig 2SA); loading controls: b-Actin (bAct). Note: the Immunoblot analysis depicted is part of the analysis as presented in Fig 6C. (C) Immunoblot analysis of two different short hairpin-based RNAi vectors targeting MK3 only (shMK3) or MK2 and MK3 simultaneously (shMK2/3); loading control Tubulin (Tub). (D) Representative DNA profiles (BrdU pulse-labeling and S-phase quantification by FACS) of TIG3/shMK3 cells corresponding to Fig 4B. (E) Quantification of DNA profiles (BrdU pulse-labeling and S-phase quantification by FACS) in TIG3/shMK3 and TIG3/con cells at approximately 1 week post-transduction. (TIF) [file pone.0118840.s002.TIF]

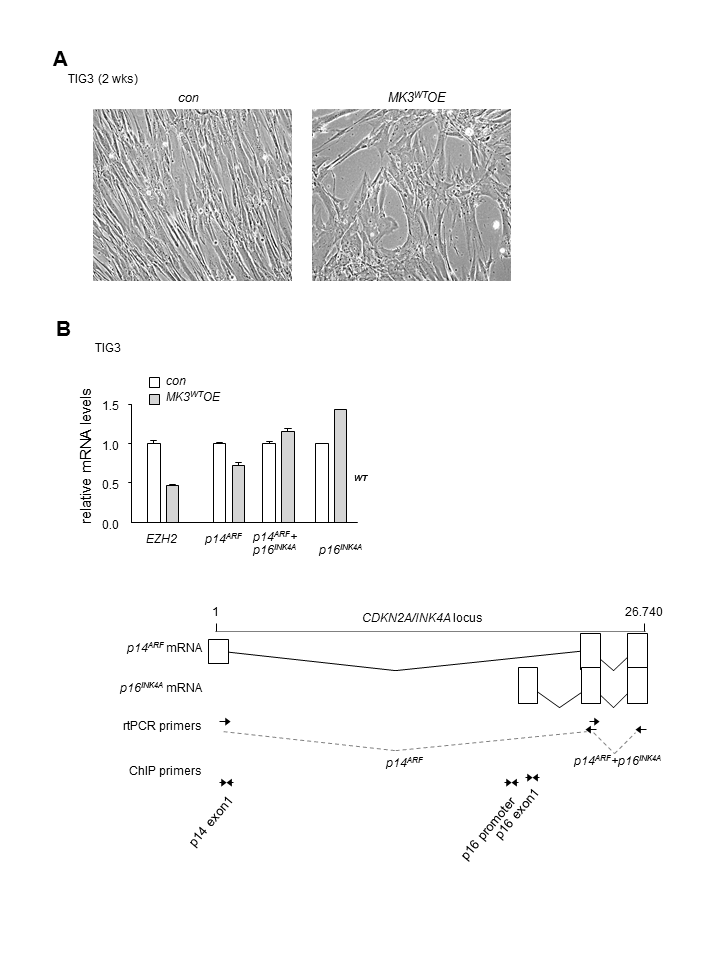

Supplement: S3 Fig — (A) Morphology of cell cultures corresponding to the chromatin immunoprecipitation (ChIP) experiments (Fig 3C); phase contrast images (right panels) confirm flat cell phenotype in TIG3/MK3 WT OE cells at the time of harvest. (B) Real-time PCR analysis of EZH2 and INK4A/ARF mRNA levels in TIG3/MK3 WT OE and control cells; bottom: schematic overview of human INK4A/ARF locus and primers used in this study. Due to overlap in mRNA sequences of p14 ARF and p16 INK4A, levels of p16 INK4A mRNA could not be directly measured by real time PCR; instead mRNA levels for p14 ARF only, and for p14 ARF + p16 INK4A were measured; p16 INK4A mRNA levels were deduced by subtraction; error bars are provided for p14 ARF and for p14 ARF + p16 INK4A measurements. (TIF) [file pone.0118840.s003.TIF]

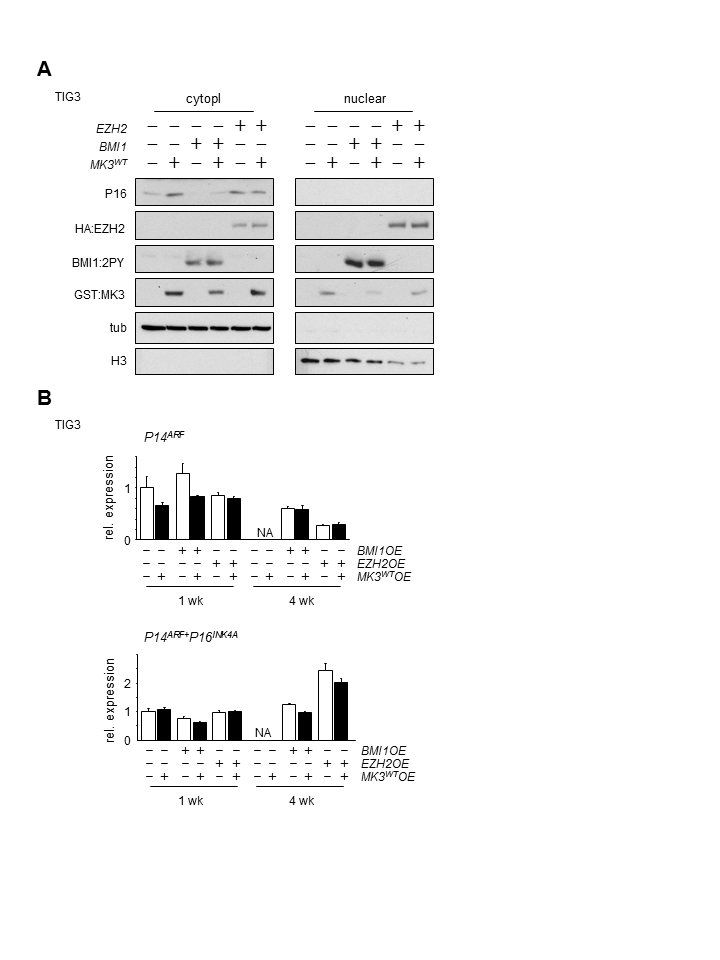

Supplement: S4 Fig — (A) Analysis of P16INK4A (P16) levels in control (con), HA-tagged EZH2 (HA:EZH2); 2PY-tagged BMI1 (BMI1:2PY) and GST-tagged MK3 (GST:MK3)-transduced TIG3 cells in the presence or absence of MK3 WT OE (GST:MK3); tubulin (tub) and histone H3 (H3) represent fractionation controls. Cytoplasmic and nuclear fractions were always loaded on the same gel for protein analysis (corresponding sections are shown separately); nuclear fractions correspond to approximately 3–4 cytoplasmic equivalents; antibodies used as indicated in figure. (B) Real-time quantitation of mRNA expression in TIG3 cells; mRNAs as indicated; note that the exon2 primer-set simultaneously detects P14ARF and P16INK4A (cf. S3 Fig) Expression data were normalized to cyclophillin expression; shRNA vectors used are indicated below the graph. (TIF) [file pone.0118840.s004.TIF]

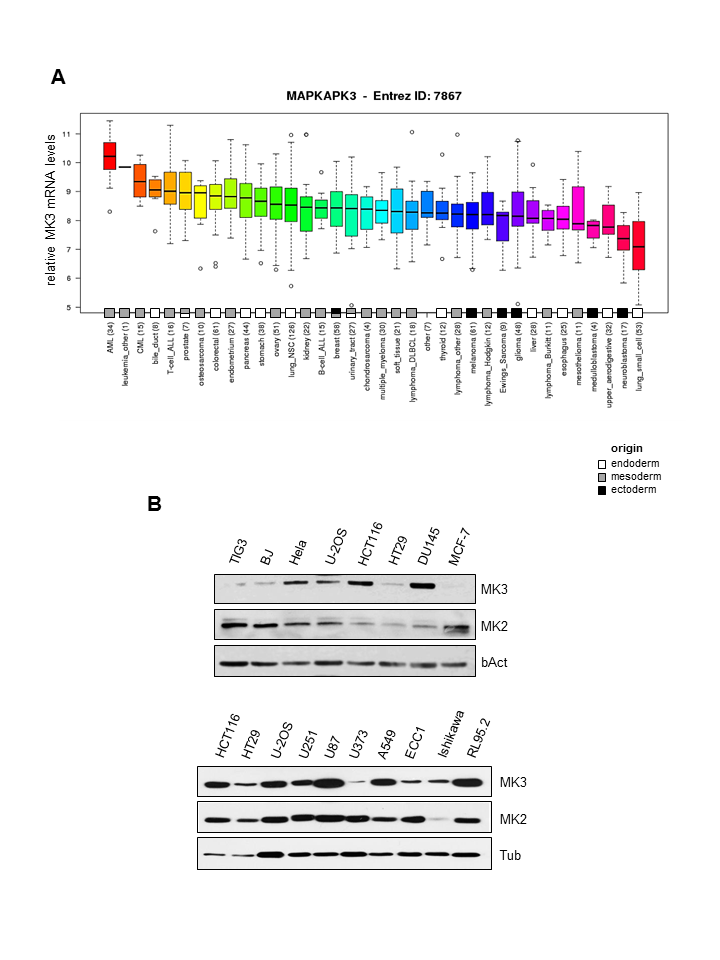

Supplement: S5 Fig — (A) Expression levels of MK3 mRNA based on analysis of nearly 1000 cancer cell lines (source: Broad-Novartis Cancer Cell Line Encyclopedia; http://www.broadinstitute.org/ccle [32]). (B) MK3 and MK2 protein expression in normal and cancer cell lines; TIG3: diploid human fibroblast; BJ2: diploid human fibroblast; HeLa: cervical carcinoma; U-2OS: osteosarcoma; HCT116, HT29: colorectal carcinoma; DU145: prostate carcinoma; MCF-7: breast carcinoma; U251, U87, U373 glioblastoma; A549: alveolar adeno-carcinoma; ECC1, Ishikawa: endometrial carcinoma; RL95-2 cervical adenosquamous carcinoma. (TIF) [file pone.0118840.s005.TIF]

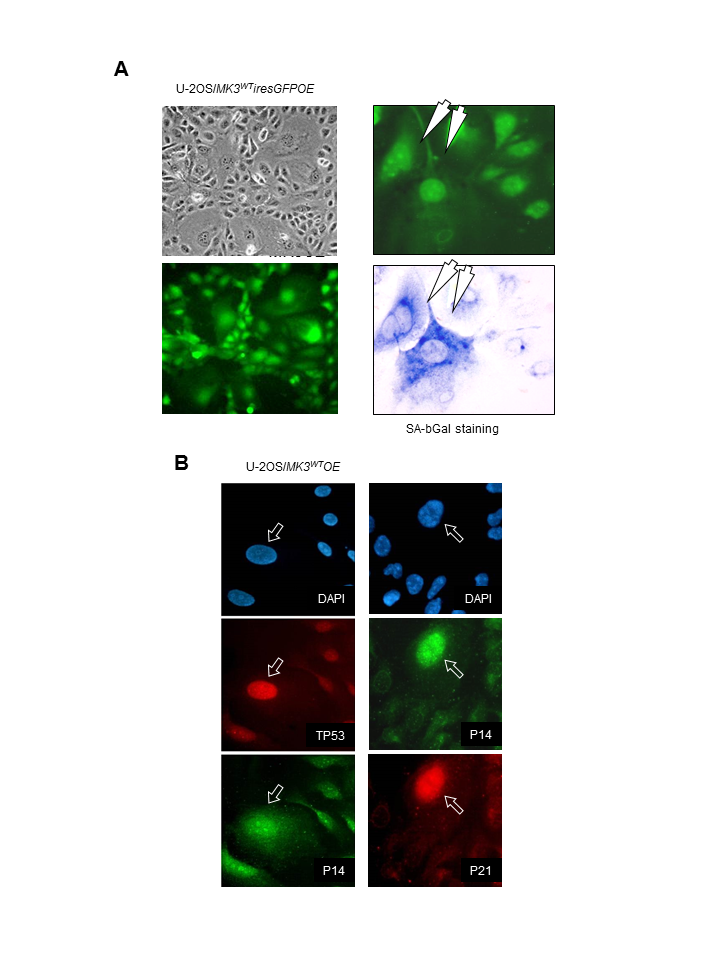

Supplement: S6 Fig — (A) Confirmation of MK3WT expression in flat U-2OS/MK3 WT iresGFP cells (upper left panel; phase contrast) transduced with a retroviral vector co-expressing GST:MK3 and GFP (lower left panel); right panels: large flat GFP-positive U-2OS cells stain positive for SA-bGal; arrows demarcate flat cells positive for GFP (upper panel) and SA-bGal (lower panel). (B) TP53 and P14ARF (P14) co-staining (left panels) or P14ARF (P14) and P21CIP1/WAF1 (P21) co-staining (right panels) in senescent U-2OS/MK3 WT OE cells; nuclei were counterstained with DAPI. (TIF) [file pone.0118840.s006.TIF]

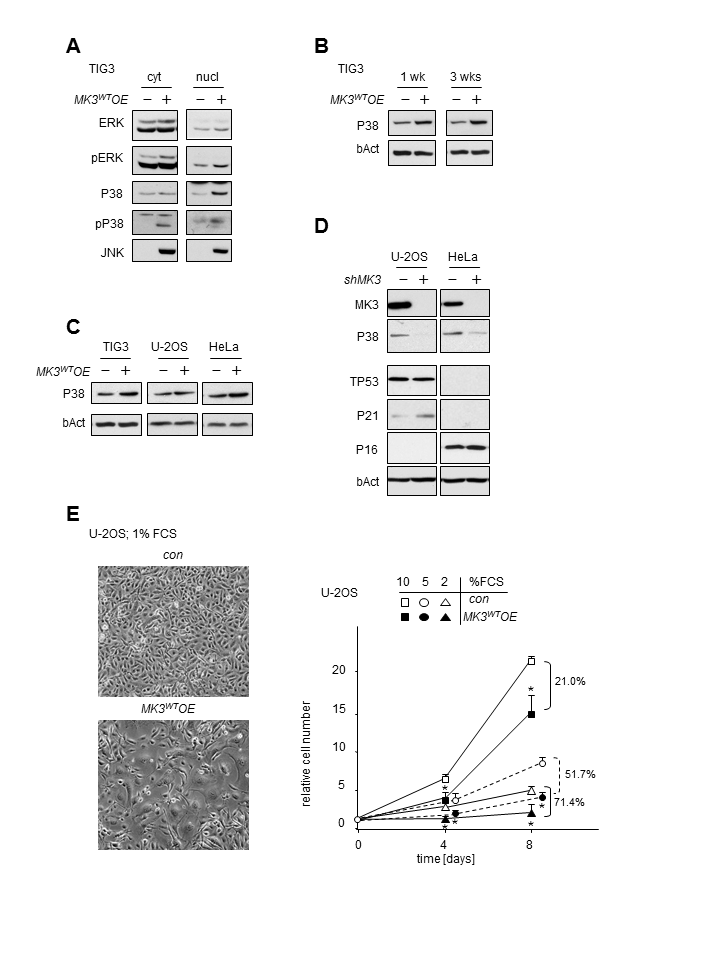

Supplement: S7 Fig — (A) Immunoblot analysis of M/SAPK (ERK, P38, JNK) and phosphorylated (pERK, pP38) levels in TIG3/MK3 WT OE (MK3 WT OE) cells versus control cells (con). (B) Immunoblot detection of sustained elevated P38 levels in TIG3/MK3 WT OE cells between 1 and 3 weeks post transduction. (C) Immunoblot detection of P38 levels in TIG3/MK3 WT OE, U-2OS/MK3 WT OE and HeLa/MK3 WT OE cells. (D) Immunoblot detection of P38, l TP53, P21cip1/waf1 (P21) and p16INK4A (P16) protein expression levels; loading controls: b-Actin (bAct). (E) Left: morphological changes in U-2OS/MK3 WT OE cultures under reduced serum conditions (1%); empty vector control cells (con). Right: quantitation of serum-deprivation on proliferative capacity of U-2OS/MK3 WT OE cells (squares: 10% FCS; circles: 5% FCS; triangles: 2% FCS; open symbols: control; filled symbols: MK3OE). Indicated (brackets) is percentage growth reduction relative to control cells at each serum concentrations. (TIF) [file pone.0118840.s007.TIF]

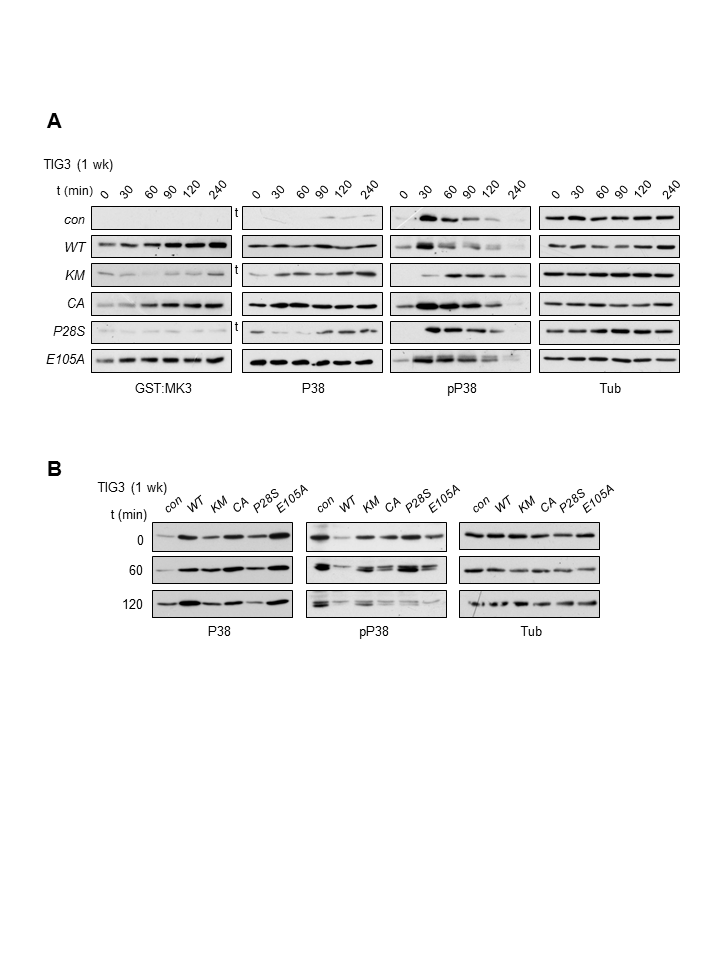

Supplement: S8 Fig — (A) Immunoblot analysis of P38 and phosphorylated P38 (pP38) levels in serum-starved/stimulated TIG3/MK3 WT OE (overexpression of wild type MK3; WT), TIG3/MK KMOE (kinase dead MK3; KM), TIG3/MK3 CA OE (constitutively active MK3; CA), TIG3/MK3 P28S OE (potential oncogenic mutation P28S; P28S) or TIG3/MK3 E105A OE (potential oncogenic mutation E105A; E105A); con represents empty retroviral vector control cells. All cells were synchronously transduced, selected, serum starved and serum/TPA stimulated. Extracts were prepared at the indicated time points (time in minutes). Loading control: Tubulin (Tub); “t” (GST:MK3 panel) refers to longer exposure time of autoradiographic film. (B) Immunoblot analysis of P38 and pP38 levels at defined time points in the same extracts as (A); extracts of all TIG3/MK3-genotypes at defined time points were loaded on the same blot to facilitate direct quantitative comparison. (TIF) [file pone.0118840.s008.TIF]

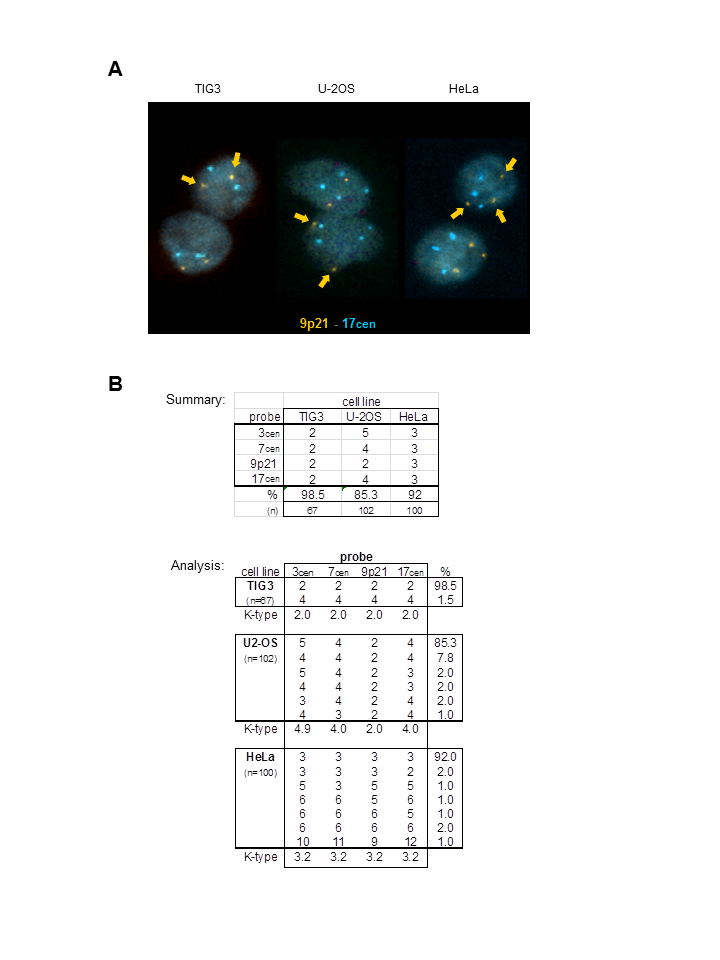

Supplement: S9 Fig — (A) Representative FISH image of TIG3 (left), U-2OS (middle) and HeLa cells (right); detection of 9p21 (CDKN2A/INK4A locus, yellow) and chromosome 17 centromeres (blue); DAPI was used to counterstain nuclei. (B) Upper table depicts summary of limited karyotype analysis; lower table depicts full analysis of TIG3 (n = 67; >98% diploid), U-2OS (n = 102; mixed polyploidy) and HeLa (n = 100; >90% triploidy at all loci investigated) nuclei for centromeres of chromosome 3, 7 and 17 and 9p21. (TIF) [file pone.0118840.s009.TIF]
