# Supplementary material for: MK3 Modulation Affects BMI1-Dependent and Independent Cell Cycle Check-Points
Source: PLoS One. 2015 Apr 8;10(4):e0118840. doi: 10.1371/journal.pone.0118840 (PMC4390245; doi:10.1371/journal.pone.0118840)
Supplement: S1 Table — (DOCX) [file pone.0118840.s010.docx]

**S1 Table.**

**Primers used for ChIP and mRNA expression analysis.**

ChIP primers

Gene

*p14ARF exon1* Forward GTGGGTCCCAGTCTGCAGTTA

Reverse CCTTTGGCACCAGAGGTGAG

*p16INK4A promoter* Forward ACCCCGATTCAATTTGGCAG

Reverse AAAAAGAAATCCGCCCCCG

*p16INK4A exon1* Forward AGAGGGTCTGCAGCGG

Reverse TCGAAGCGCTACCTGATTCC

*p15 exon1* Forward GGAACCTAGATCGCCGATGTAG

Reverse TGTTTTACGCGTGGAATGCAC

*HOXA10* Forward CCCGAGCTGATGAGCGAGTC

Reverse GCCAAATTATCCCACAACAATGTC

*HOXA11* Forward AATCTATCCCCATCCTTAGCAGG

Reverse TTGTCAATTTCAACATCGGGTC

Primers quantitative expression analysis.

Gene

*EZH2* Forward GGGACAGTAAAAATGTGTCCTGC

Reverse TGCCAGCAATAGATGCTTTTTG

*p14ARF* Forward CCCTCGTGCTGCTGATGCTACTG

Reverse CCCATCATCATGACCTGGTCTT

*p14ARF+p16INK4A* Forward GAAGGTCCCTCAGACATCCCC

Reverse CCCTGTAGGACCTTCGGTGAC

*cyclophillin A* Forward TTCCTGCTTTCACAGAATTATTCC

Reverse GCCACCAGTGCCATTATGG

**shRNA target sequences.**

*shMK3* CGGCAAAGTGCTGGAGTGC

*shBMI1* GAATGGTCCACTTCCATTG

*shPHC2* GTTCAAGCGTCCAAGCGC
